# The *Acer truncatum* genome provides insights into nervonic acid biosynthesis

**DOI:** 10.1111/tpj.14954

**Published:** 2020-10-02

**Authors:** Qiuyue Ma, Tianlin Sun, Shushun Li, Jing Wen, Lu Zhu, Tongming Yin, Kunyuan Yan, Xiao Xu, Shuxian Li, Jianfeng Mao, Ya‐nan Wang, Shuangxia Jin, Xing Zhao, Qianzhong Li

**Affiliations:** ^1^ Institute of Leisure Agriculture Jiangsu Academy of Agricultural Sciences Jiangsu Key Laboratory for Horticultural Crop Genetic Improvement Nanjing 210014 China; ^2^ Novogene Bioinformatics Institute Beijing 100083 China; ^3^ Co‐Innovation Center for Sustainable Forestry in Southern China College of Forestry Nanjing Forestry University Nanjing 210037 China; ^4^ The Southern Modern Forestry Collaborative Innovation Center Nanjing Forestry University Nanjing 210037 China; ^5^ Beijing Advanced Innovation Center for Tree Breeding by Molecular Design College of Biological Sciences and Technology Beijing Forestry University Beijing 100083 China; ^6^ National Key Laboratory of Crop Genetic Improvement Huazhong Agricultural University Wuhan Hubei 430070 China

**Keywords:** *Acer truncatum*, nervonic acid, *de novo* assembly, very long‐chain monounsaturated fatty acid, KCS

## Abstract

*Acer truncatum* (purpleblow maple) is a woody tree species that produces seeds with high levels of valuable fatty acids (especially nervonic acid). However, the lack of a complete genome sequence has limited both basic and applied research on *A. truncatum*. We describe a high‐quality draft genome assembly comprising 633.28 Mb (contig N50 = 773.17 kb; scaffold N50 = 46.36 Mb) with at least 28 438 predicted genes. The genome underwent an ancient triplication, similar to the core eudicots, but there have been no recent whole‐genome duplication events. *Acer yangbiense* and *A. truncatum* are estimated to have diverged about 9.4 million years ago. A combined genomic, transcriptomic, metabonomic, and cell ultrastructural analysis provided new insights into the biosynthesis of very long‐chain monounsaturated fatty acids. In addition, three *KCS* genes were found that may contribute to regulating nervonic acid biosynthesis. The *KCS* paralogous gene family expanded to 28 members, with 10 genes clustered together and distributed in the 0.27‐Mb region of pseudochromosome 4. Our chromosome‐scale genomic characterization may facilitate the discovery of agronomically important genes and stimulate functional genetic research on *A. truncatum*. Furthermore, the data presented also offer important foundations from which to study the molecular mechanisms influencing the production of nervonic acids.

## Significance Statement

Here, we provide the first report on the *Acer truncatum* genome. Our work provides extensive genetic resources necessary for very long‐chain monounsaturated fatty acid biosynthesis.

## INTRODUCTION

**Figure 1:**
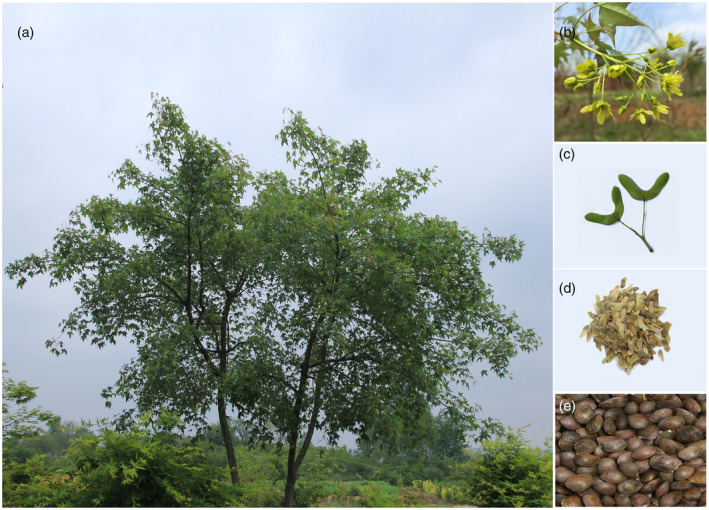
Photographs of *A. truncatum*. (a) Mature tree. (b) Flowers. (c) Immature fruits. (d) Mature fruits. (e) Seeds.

Purpleblow maple (*Acer truncatum* Bunge, 2n = 2x = 26) is a diploid monoecious tree species of the family Aceraceae. It is a versatile oil‐producing woody tree that is a native species widely distributed in northern China, Korea, and Japan, but it has also been identified in Europe and North America (More *et al*., 2003; Guo *et al*., 2014). In China, this tree species is referred to as ‘yuan bao feng’ because of its gold ingot‐shaped fruits (Figure 1). It has historically been used for landscaping. Potential further uses of the various tree parts have been revealed through years of research. For example, *A. truncatum* leaves are used to produce health‐promoting tea and folk medicines for treating cerebrovascular diseases and angina pectoris (Ma *et al*., 2005) because of their substantial abundance in tannins, flavonoids, and chlorogenic acid (Lingguang *et al*., 2017). Moreover, *A. truncatum* seed oil is extracted by pressing the kernels, and is composed mainly of triacylglycerols with approximately 90% unsaturated fatty acids (including oleic acid: 25.8%, linoleic acid: 37.3%, and nervonic acid [NA]: 5.5%) (Wang *et al*., 2006; Liu *et al*., 2013).

NA (24:1Δ15, *cis*‐15‐tetracosenoic acid, *n*‐9) is a very long‐chain monounsaturated fatty acid (VLCFA) that was first discovered in the brain of sharks. It has been detected mainly in the brain and nervous system tissues, where it is considered an important component that contributes to myelinated nerve fiber biosynthesis (Poulos, 1995; Merrill *et al*., 1997). In humans, abnormal NA levels can lead to several neurological disorders or mental illnesses, including schizophrenia, psychosis, and attention deficit disorder (Chen *et al*., 2004; Pamplona *et al*., 2005; Tanaka *et al*., 2007; Amminger *et al*., 2012). NA‐containing oils have been important agents for preventing and treating neurological disorders and diseases. The beneficial attributes of NA include its ability to inhibit the reverse transcriptase activity of human immunodeficiency virus type‐1 (Kasai *et al*., 2002). Thus, NA oils are important bioactive lipid supplements for promoting human health. Although the availability of NA sources is limited, the seeds of several plant species reportedly contain relatively large amounts of NA within storage lipids (e.g., *Cardamine graeca*, *Malania oleifera*, *A. truncatum*, *Lunaria annua*, *Borago officinalis*, and *Tropaeolum speciosum*) (Bettger *et al*., 2001; Wang and Wang, 2005; Guo *et al*., 2009; Taylor *et al*., 2009; Yang *et al*., 2018). Additional natural plant resources rich in NAs are needed for large‐scale NA production to satisfy the high market demand, but these plant species may be of limited utility because of issues related to their NA contents and growth adaptability. *A. truncatum* might be a viable NA resource for commercial production, with features such as rapid growth, wide geographic distribution, and high adaptability. In 2011, *A. truncatum* seed oils were certified as a new food resource by the Ministry of Health of the People’s Republic of China, with potentially important implications for the fields of food and medicine.

NA is synthesized in the endoplasmic reticulum by a process involving the following four key enzymes: 3‐ketoacyl CoA synthetase (KCS), 3‐ketoacyl CoA reductase (KCR), 3‐hydroxyacyl CoA dehydratase (HCD), and *trans*‐2,3‐enoyl‐CoA reductase (ECR) (Fehling and Mukherjee, 1991; Samuels *et al*., 2008; Wang *et al*., 2018). KCS is a key rate‐limiting enzyme that determines the tissue specificity and the substrate for fatty acid elongation (Mietkiewska *et al*., 2007; James *et al*., 1995; Mietkiewska *et al*., 2007). Previous studies revealed that NA levels were raised in the seed oil of *KCS*‐expressing transgenic plants and NA was found among the fatty acids of *KCS*‐expressing transgenic yeast (Taylor *et al*., 2009; Dongxin *et al*., 2015).

The genus *Acer* L. comprises more than 200 species that grow in China, including many with considerable medicinal, ornamental, and economic value (Xu, 1998). To date, there is a lack of a fully sequenced genomes among the species in this genus. In the present study, we completed a *de novo* sequence and assembly of the genome of *A. truncatum*. An analysis of this genome revealed features such as high heterozygosity and highly similar repeats. Moreover, examination of the transposable elements (TEs) of *A. truncatum* suggested a TE burst occurred 1–2 million years ago (Mya), which is relatively recent in evolution, while *A. truncatum* diverged from *Acer yangbiense* about 9.4 Mya. Comprehensive comparisons between the *A. truncatum* and *A. yangbiense* genomes were also conducted, including the identification of centromeric regions and expanded and contracted gene families. Moreover, comparative transcriptomic and metabonomic analyses were completed to obtain new insights into the characterization of the late embryogenesis abundant (*LEA*) gene family and NA biosynthesis. The genome sequence represents a valuable resource for genetic studies and for accelerating the breeding of new lines with increased production of bioactive compounds, particularly NA.

## RESULTS

### Genome sequencing, assembly, and annotation

**Figure 2:**
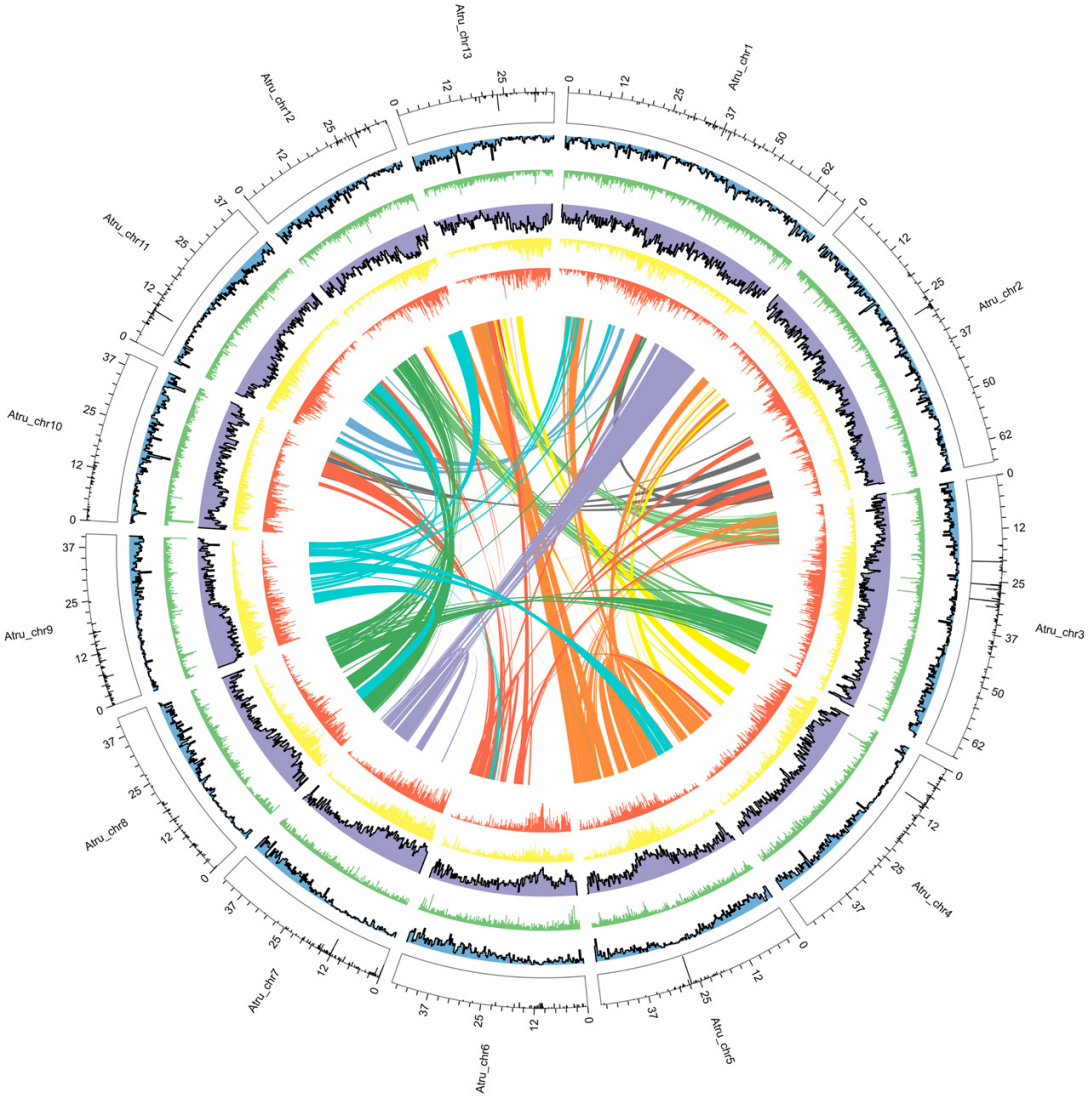
Circos plot of the genomic features of *Acer truncatum*. Concentric circles, from innermost to outermost, show: intra‐genome collinear blocks connected by curved lines, LTR‐*Gypsy* density (red), LTR‐*Copia* density (yellow), transposon element density (purple), tandem repeats density (green), and gene density (blue). The outermost circle displays the narrow peaks which represent centromeric regions. All distributions are drawn in a window size of 300 kb, chromosomes_units = 500 000.

The *A. truncatum* genome was sequenced using a combination of PacBio single‐molecule long reads and Illumina short reads. We improved the assembly by adding 10× Genomics linked reads. On the basis of a K‐mer analysis, the *A. truncatum* genome size was estimated to be 653.44 Mb, with a heterozygosity of 1.12% (Table S1 and Figure S1). High‐quality consensus sequences were assembled from PacBio long reads (115.85 × coverage) (Table S2) with the Falcon program (Chin *et al*., 2016), after which errors in the assembly were corrected with Illumina short reads (180.48 × coverage) and the Pilon program (Walker *et al*., 2014). We generated a genome assembly comprising 628.84 Mb with a contig N50 of ~773.17 kb. The 10 × Genomics data (124.79 × coverage) were used to scaffold the genome with the FragScaff program (Mostovoy *et al*., 2016). The chromosome‐scale scaffolds were finally assembled based on Hi‐C data (72.21 Gb; Figure S2). A total of 13 long super‐scaffolds (hereafter denoted as pseudochromosomes) were generated, representing 99.44% (Table S3) of the final genome assembly with a total genome size of 633.28 Mb and a scaffold N50 of 46.36 Mb (Figure 2 and Table S4). Regarding the constructed pseudochromosomes, the Hi‐C interaction matrices displayed a distinct diagonal pattern for the intrachromosomal interactions (Figure S2), indicating that most of the contigs were accurately oriented on the pseudochromosomes. The mapping rate reached 97.24% when we aligned the Illumina reads to the genome assembly (Table S5). In addition, the completeness of the *A. truncatum* genome assembly was evaluated with the Core Eukaryotic Genes Mapping Approach (CEGMA) and the Benchmarking Universal Single‐Copy Orthologs (BUSCO) assessment. Accordingly, 239 of 248 core eukaryotic genes (96.37%) and 1342 complete gene models among 1440 conserved genes (93.2%) were identified based on the CEGMA and BUSCO analyses, respectively (Tables S6 and S7). In addition, 100 924 (95.16%) of unigenes (>500 bp) assembled by Trinity mapped to our genome assembly (Table S8) and the long terminal repeat (LTR) Assembly Index (LAI) score was 16.4 (Ou *et al*., 2018). These results for the evaluation of genome quality verified that our genome assembly was accurate and complete at the chromosome scale.

We applied a combination of *de novo*, homology, and transcript‐based approaches to predict the genes in the *A. truncatum* genome. Approximately 48 Gb RNA sequencing data were generated from five libraries representing major tissue types (root, stem, young leaf, flower bud, and mature seed). In all, 28 438 genes were predicted, with average coding sequence and intron lengths of 1111.6 and 636 bp, respectively. Of these genes, 28 290 genes (99.48%) were anchored to 13 pseudochromosomes (Table S4). Moreover, 28 023 (98.5%) were similar to known genes and were functionally annotated in at least one of the following databases: NR, Swiss‐Prot, KEGG, and InterPro (Table S9). Of the predicted gene models, 21 579 genes (75.87%) contained Pfam domains and 25 432 genes (89.43%) were assigned to Gene ontology (GO) categories. Additionally, non‐coding RNA sequences were predicted in the *A. truncatum* genome, including 1345 miRNAs, 744 tRNAs, 368 rRNAs, and 868 snRNAs, with an average length of 132.48, 75.11, 169.31, and 113 bp, respectively (Table S10).

### Repetitive content and structure of centromeres

Using a combination of approaches, the repetitive elements were determined to represent approximately 391.05 Mb (61.75%) of the assembled *A. truncatum* genome (Tables 1 and S11). Most of these repetitive elements were TEs (approximately 379.62 Mb; 59.95%; Table 1). Similar to other plants, LTRs were the predominant retrotransposon in *A. truncatum* (approximately 287.49 Mb; 45.40%; Table S11) and most of the LTRs were *Copia* and *Gypsy*. The retroelements with the highest copy numbers were the *Tork* subfamily members (2200) of the *Ty1/Copia* family, followed by the *Tat* subfamily members (1789) of the *Ty3/Gypsy* family. Similar to *A. truncatum*, *Tork* (668) also represented the highest copy numbers in the *Ty1/Copia* subfamilies of *Camellia sinensis*. However, in the *A. yangbiense* and *M. oleifera* genomes, *Sire* has the highest copy numbers, with 6624 and 8373 copies, respectively. *Athila* (751), *Tat* (1831), and *Del* (10 005) were the *Ty3/Gypsy* subfamilies with the highest copy numbers in *C. sinensis*, *A. yangbiense*, and *M. oleifera*, respectively (Table S12). An examination of the recent TE activities among *A. truncatum*, *A. yangbiense*, *C. sinensis*, and *M. oleifera* revealed a recent burst in *A. truncatum* and *A. yangbiense* around 1–2 Mya, whereas a substantial abundance of TEs was inserted into the *M. oleifera* genome about 4–5 Mya (Figure S3). These results provide important information regarding the genome evolution of Sapindales species.

**Table 1:** Global statistics of *Acer truncatum* genome assembly and annotation

	Number	Size
Assembly		
Estimated genome size		653.44 Mb
Scaffolds	34	633.28Mb
N50 of scaffolds	6	46.36 Mb
Longest scaffolds		70.31 Mb
Contigs	1453	628.84 Mb
N50 of contigs	239	773.17 kb
Longest contigs		4.57 Mb
Pseudochromosomes	13	629.76 Mb
Annotation		
Repetitive sequences	61.75%	391.05Mb
Transposable element	59.95%	379.62 Mb
Protein‐coding genes	28 438	
Mean gene length		3457.58 bp
Mean coding sequence length		1111.28 bp
Mean intron length		636.49 bp
Mean exon length		237.15 bp
Non‐coding RNAs	3325	394, 462 bp

We also identified centromeric regions in *A. truncatum* and *A. yangbiense* by searching highly clustered tandem repeats (VanBuren *et al*., 2015). Most chromosomes of *A. truncatum* (except Chr10) and *A. yangbiense* (except Chr5) had narrow peaks (Figures 2 and S9), representing clustered satellites with a base monomer length of 161 bp (GC content: 53%) and 160 bp (GC content: 41.25%), respectively (Table S13). These clustered satellites were located in *Gypsy*‐ and *Copia‐*rich regions, also indicating they are putative centromeric regions of *A. truncatum* and *A. yangbiense* chromosomes. The putative centromeric regions in the *A. truncatum* chromosomes verified the completeness of our genome assembly.

### Comparative genomic analysis

To investigate the relationship between gene families and specific *A. truncatum* traits, we clustered the orthologs of *A. truncatum* and 14 other sequenced plant species, yielding 18 616 gene groups, including 109 single‐copy gene families (Figure S4). We analyzed the phylogenetic relationships among *A. truncatum* and 14 other plant species with 109 single‐copy orthologs. The results revealed that *A. truncatum* clustered with *A. yangbiense* and both species were located close to *C. sinensis*, which was the expected result, as the three species belong to the order Sapindales (Xu *et al*., 2013). Moreover, an examination of divergence times indicated that *A. truncatum* diverged from *A. yangbiense* and *C. sinensis* about 9.4 and 68.6 Mya, respectively, after the common ancestor of both Sapindales species diverged (approximately 92.8 Mya) (Figure S5). Meanwhile, to determine the phylogenetic position of *A. truncatum* in Aceraceae, 17 complete chloroplast genome sequences of Aceraceae species were obtained (Tables S14) and used to construct phylogenetic trees (Figures S6 and S7). It showed that *A. truncatum* is closely related to *Acer catalpifolium* and *Acer miaotaiense*.

**Figure 3:**
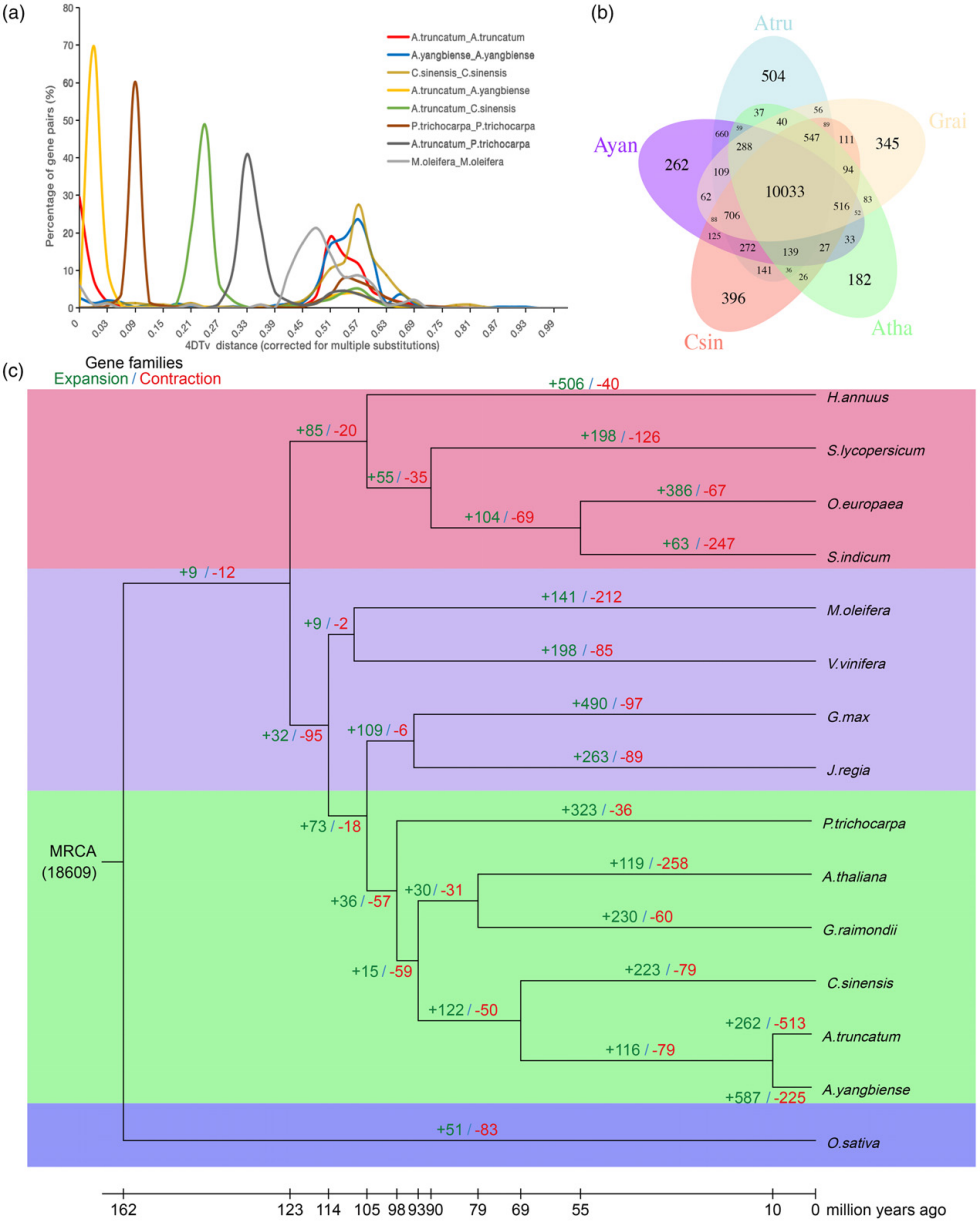
Genome evolution and comparative analysis of the *Acer truncatum* genome. (a) WGD analysis of *A. truncatum*, where the *x*‐axis indicates 4DTv distribution of transversion substitutions at fourfold degenerate sites. (b) Venn diagram of *A. truncatum* (Atru), *C. sinensis* (Csin), *G. raimondii* (Grai), *A. thaliana* (Atha), and *A. yangbiense* (Ayan). Each number in the diagram is the number of gene families within a group. (c) Gene family expansion and contraction among 15 plants. The green and red values above each branch represent the expanded and contracted gene families after the diversification from the most common ancestor, respectively.

A further comparison of *A. truncatum*, *C. sinensis*, *Gossypium raimondii*, *Arabidopsis thaliana*, and *A. yangbiense* revealed 10 033 gene clusters shared by the five malvid species. In addition, we found 504 gene families that were unique to the *A. truncatum* genome when compared with the other four genomes (Figure 3(b)). An investigation of gene family evolution indicated that 262 gene families expanded in *A. truncatum*, whereas 513 gene families contracted (Figure 3(c), Tables S15–S18). The number of expanded gene families (587) was higher than that of contracted gene families (225) in *A. yangbiense* (Tables S19 and S20). The expanded gene families of two genomes were both mapped to the Kyoto Encyclopedia of Genes and Genomes (KEGG) pathways. In the *A. truncatum* genome, we observed significant enrichment (*P* < 0.05) in environment adaptation, flavonoid biosynthesis, phenylpropanoid biosynthesis, fatty acid elongation, and linoleic acid and nitrogen metabolism (Tables S18). Compared with the *A. truncatum* genome, the *A. yangbiense* genome is significantly enriched (*P* < 0.05) in environment adaptation, cell cycle, cyanoamino acid metabolism, phenylpropanoid biosynthesis, and pentose and glucuronate interconversions (Tables S21). These functions might be related to plant defense responses and the production of secondary metabolites.

On the basis of the accumulated rate at fourfold synonymous third‐codon transversion (4DTv) of the duplicate gene pairs, the peak values in *A. truncatum*, *A. yangbiense*, and *C. sinensis* were 0.51, 0.57, and 0.57, respectively (Figure 3(a)). These similar peaks indicated that they underwent the same whole‐genome duplication (WGD), the γ event. Moreover, we identified 159 syntenic blocks comprising 3891 collinear genes (13.75% of 28 290 genes) in *A. truncatum*. A total of 396 syntenic blocks were detected between *A. truncatum* and *C. sinensis* with 1:1 syntenic patterns (cscore >0.7), and a total of 171 syntenic blocks were detected between *A. truncatum* and *A. yangbiense* (Figures S8–S10). The results also showed that *A. truncatum* lacks a recent independent WGD in its evolutionary history, similar to *A. yangbiense* (Yang *et al*., 2019) and *C. sinensis* (Xu *et al*., 2013); only an ancient γ event occurred in *A. truncatum*.

### Analysis of LEA protein family in *A. truncatum*

Abiotic stresses, including drought, extreme temperatures, and salinity, restrict plant growth and development. *A. truncatum* is drought‐tolerant and can survive in severely barren environments. In our study, 433 genes and 132 transcription factors (TFs) related to drought resistance were identified in *A. truncatum* and a comparative analysis with five other plant species with annotated genomes was also completed (*A. thaliana*, *C. sinensis*, *M. oleifera*, *A. yangbiense*, and *Solanum lycopersicum*) (Tables S22 and S23). The results suggested that a set of genes related to drought resistance had expanded, including members of the *MSR*, *AQP1*, *LEA*, *LEW*, *TIP*, and *SUS* gene families (Table S22). These expanded gene families might contribute to enhanced resistance in *A. truncatum*. The LEAs have received increasing attentions in recent years and have been shown to play important roles in the responses against various stresses, including drought and salinity (Hincha and Thalhammer, 2012; Gao and Lan, 2016; Magwanga *et al*., 2018).

We analyzed the characteristics of the *LEA* gene family in *A. truncatum*. The results showed that LEAs expanded to 82 genes in *A. truncatum*, which is more than the number of LEAs detected in the *A. thaliana* (50), *C. sinensis* (72), *M. oleifera* (55), *A. yangbiense* (68), and *S. lycopersicum* (74) genomes (Table S22). From the results of a phylogenetic analysis, the *A. truncatum* *LEA* genes were further classified into the following seven groups: *LEA1* (4), *LEA2* (43), *LEA3* (6), *LEA4* (8), *LEA5* (3), *SMP* (7), and *dehydrin* (11) (Figure S11). The *LEA* genes are distributed across every pseudochromosome except 11. The majority of genes are located on pseudochromosomes 1, 3, and 5 (Figure S12). In previous studies, LEA proteins were found to be expressed in seedlings, stems, roots and other organs throughout all developmental stages (Shao *et al*., 2005; Du *et al*., 2013; Pedrosa *et al*., 2015). As shown in Figure S13, the expression of LEAs in *A. truncatum* was very diverse in five organs. Most of the *LEA2* group members were highly expressed in the root or stem. However, the *SMP* and *dehydrin* members were most highly expressed in the seed. Ten *LEA* genes from different groups were selected to determine their expression pattern by real‐time PCR (RT‐PCR) analysis. The results were mostly consistent with the RNA‐seq data (Figures S13–S15). Moreover, the expression levels of most genes gradually increased in the later stages of seed development (Figure S14). Our results will pave the way for future functional analyses to unravel the role of *LEA* genes in drought resistance in *A. truncatum*.

### Analysis of fatty acid biosynthesis in *A. truncatum*

**Figure 4:**
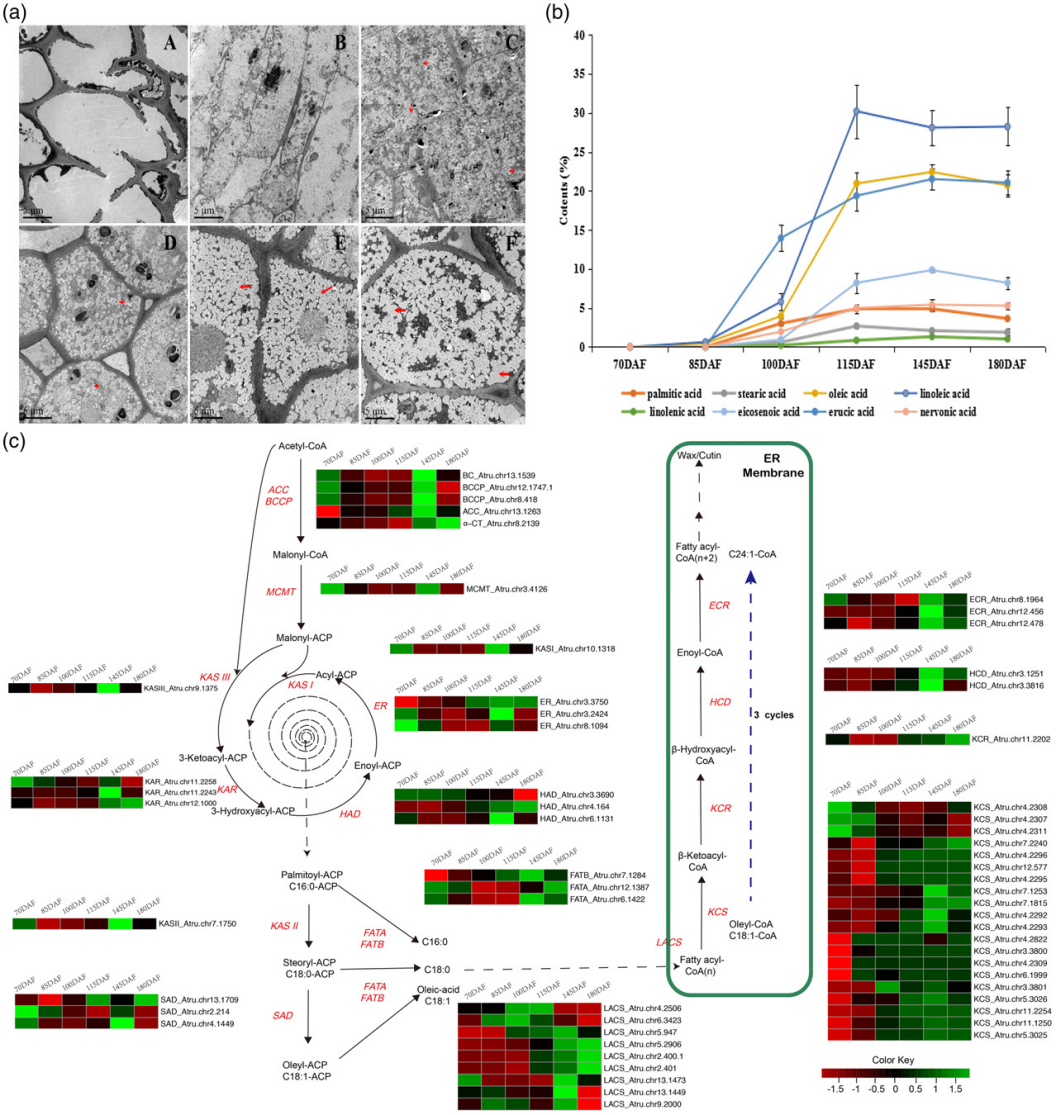
Detailed biosynthetic pathways of VLCFA in *A. truncatum*. (a) Oil bodies in seed cells, as observed by electron microscopy, at (A) 70, (B) 85, (C) 100, (D) 115, (E) 145, and (F) 180 DAF. (b) The extraction and determination of fatty acid contents in *A. truncatum* seeds after flowering. The *x*‐axis indicates the time after flowering (in days) and the *y*‐axis indicates the oil content (in %). (c) Different columns of each heat map indicate different numbers of days of seed development (70–180 DAF). Gene expression levels (log_10_(RPKM + 1)) in five tissues are represented by color gradation. Gene expression with RPKM ≤ 1 was set to 0 after log_10_ transformation. Genes with more than one homologous gene are represented by equally colored horizontal stripes and are termed from top to bottom. ACC: acetyl‐CoA carboxylase; BC: biotin carboxylase; BCCP: biotin carboxyl carrier protein; ER: enoyl‐ACP reductase; HAD: hydroxyacyl‐ACP dehydrase; KAR: ketoacyl‐ACP reductase; KASI, II, and III: ketoacyl‐ACP synthase I, II, and III; MCMT: malonyl‐CoA:acyl carrier protein (ACP) malonyltransferase; α‐CT: α‐carboxyltransferase; SAD: stearoyl‐ACP desaturase; FATA (B): fatty acyl thioesterase A (B); LACS: long‐chain acyl‐CoA synthetase; KCS: ketoacyl‐CoA synthase; KCR: ketoacyl‐CoA reductase; HCD: hydroxyacyl‐CoA dehydrase; ECR: enoyl‐CoA reductase.

Fatty acid biosynthesis, which is one of the major steps involved in the production of complex oils, is completed via the activities of fatty acid synthases, elongases, desaturases, and carboxylases. In our study, the combined results of genomic, transcriptomic, cell ultrastructural, and gas chromatography–mass spectrometry analyses provided some new insights regarding this biosynthesis pathway in *A. truncatum*. The fatty acid contents and cell ultrastructures were analyzed during six seed development stages (i.e., 70, 85, 100, 115, 145, and 180 days after flowering [DAF]). Fatty acids were undetectable at 70 DAF, but the oleic acid (18C:1) and linoleic acid (18C:2) contents increased rapidly from 85 DAF (0.46% and 0.63%, respectively) to 115 DAF (20.93% and 30.21%, respectively) (Figure 4(b)). In contrast, the other fatty acids, including erucic acid (22C:1) and NA (24C:1), apparently accumulated after 85 DAF. NA was undetectable at 85 DAF but increased to 1.96% at 100 DAF (Figure 4(b)). Lipids that accumulate in seeds are generally stored in oil bodies (Gu *et al*., 2017), The lipid bodies of the cotyledons during various seed development stages were examined using the cell ultrastructural analysis. The results confirmed that the key period for oil production was between 85 DAF (no oil bodies) and 100 DAF (oil bodies detected) (Figure 4(a)). These observations are consistent with the results of our NA content analysis. Moreover, the lipid bodies gradually increased in size and filled the endosperm cells over the subsequent 80 days (Figure 4(a)).

**Figure 5:**
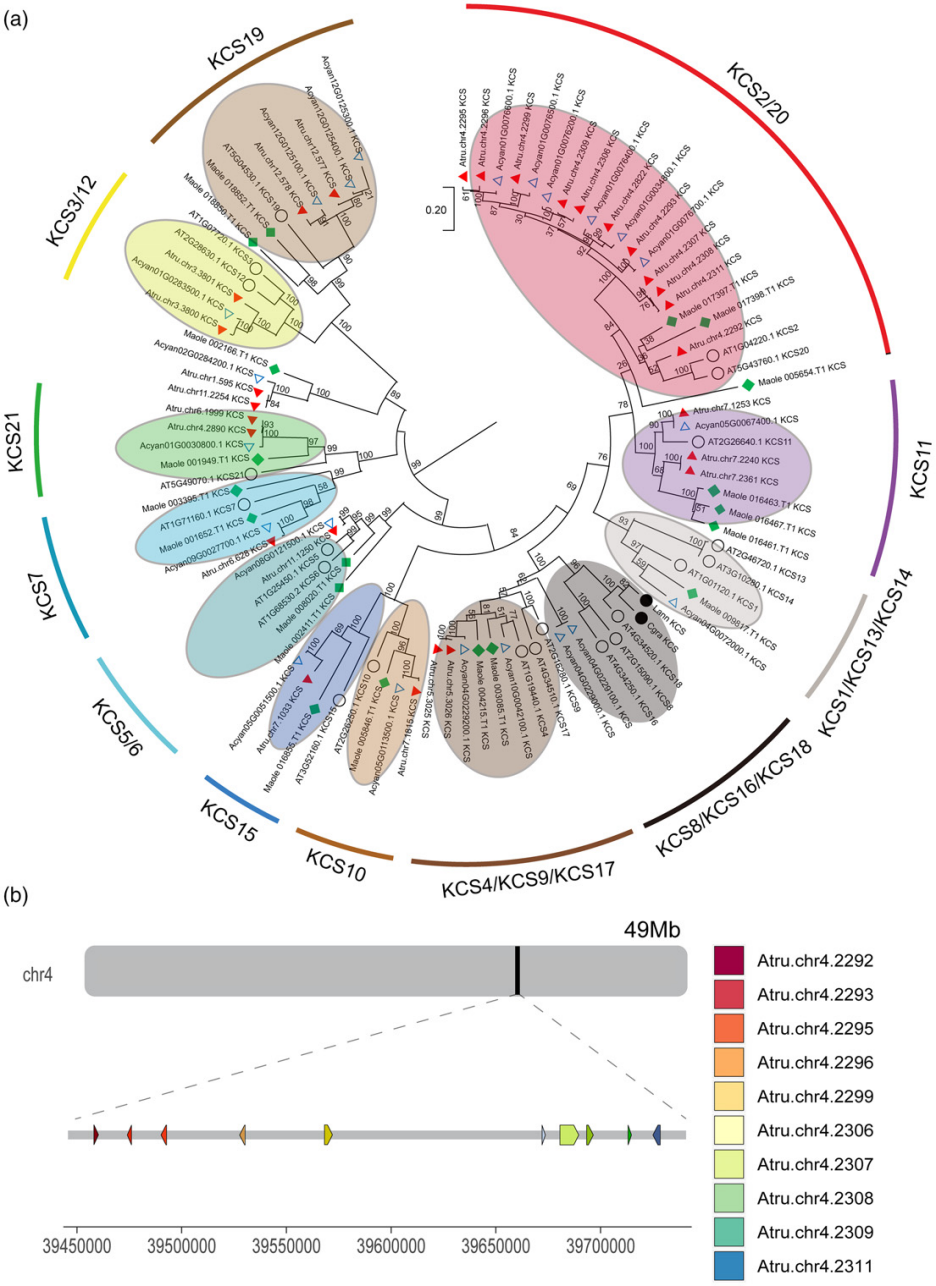
The phylogenetic tree and distribution of *KCS* genes based on protein sequences of *Acer truncatum*. The phylogenetic tree and distribution of *KCS* genes. (a) The maximum likelihood tree of *KCS* genes. *A. truncatum* (red triangle), *A. yangbiense* (write triangle), *M. oleifera* (blue square), *A. thaliana* (write circle), *C. graeca* (Cgra), and *L. annua* (Lann) genes are shown in the tree with corresponding gene ID nomenclature. (b) the distribution of 10 *KCS* genes on chromosome 4 of *A. truncatum*. Each arrow represents a *KCS* gene.

To elucidate the molecular mechanisms underlying VLCFA production, especially NA biosynthesis, in *A. truncatum*, we identified 68 genes that are important for the VLCFA biosynthesis pathway (Table S24). There were some differences in the number of duplications of genes encoding enzymes involved in the fatty acid biosynthesis pathway (Figure 4(c)). A few enzyme‐encoding gene families related to lipid metabolism (*ECR*, *ER*, *HAD*, *KAR*, *SAD*, *LACS*, and *KCS*) underwent more than three duplications (Figure 4(c) and Table S20). NAs (C24:1) can be synthesized from oleyl‐CoA (C18:1‐CoA) by the four enzyme‐catalyzed reactions of the elongation cycle on the endoplasmic reticulum via three additions of C_2_ moieties (Yang *et al*., 2018). In the *A. truncatum* genome, 34 genes were predicted to affect the four reactions of the elongation cycle, including three genes encoding ECR, two encoding HCD, one encoding KCR, and 28 encoding KCS (Figure 4(c) and Table S24). Among these enzymes, KCS is considered to be the rate‐limiting enzyme during fatty acid elongation because it determines the substrate and tissue specificities. Previous studies confirmed that the *KCS* gene is important for NA biosynthesis (Taylor *et al*., 2009; Dongxin *et al*., 2015). In the current study, we determined that the *A. truncatum KCS* gene family expanded to 28 genes. A phylogenetic analysis of *A. truncatum*, *A. yangbiense*, *A. thaliana*, and *M. oleifera* showed that their *KCS* gene families consist of 28, 22, 21, and 19 members, respectively. The results indicated that 10 of the *A. truncatum KCS* genes and 6 *A. yangbiense KCS* genes were classified in the same group, and closely related to the three clustered genes (*KCS_Maole_016461.T1*, *KCS_Maole_016463.T1*, *KCS_Maole_016467.T1*). Xu *et al*. (2019) identified three genes that were duplicated and predicted to be important in regulating the VLCFA biosynthesis pathway in *M. oleifera*. Those genes were also closely related to three *A. thaliana KCS* genes (*KCS11*, *KCS2*, and *KCS20*) (Figures 5(a) and S16). Interestingly, the 10 *A. truncatum KCS* genes underwent sequential tandem duplications and were clustered in the 0.27‐Mb region of pseudochromosome 4 (Figure 5(b)). In our study, the expression levels of most of the *KCS* genes were high during the early seed development stages and then gradually decreased. However, the expression patterns of *Chr4.2308.KCS*, *Chr4.2307.KCS*, and *Chr4.2311.KCS* differed from those of the other genes analyzed (Figure S17), with expression level trends that were generally consistent with the accumulation of NA (Figure 4(c)). Interestingly, they are all clustered in a small branch (Figure 5(a)). To confirm the expression level trends at six seed development stages, *Chr4.2308.KCS*, *Chr4.2307.KCS*, and *Chr4.2311.KCS*, including two randomly selected *KCS* genes (*Chr4.2309.KCS* and *Chr4.2822.KCS*) in the clustered group, were used to confirm the expression patterns by RT‐PCR analysis. We found that the expression levels of *Chr4.2308.KCS*, *Chr4.2307.KCS*, and *Chr4.2311.KCS* were all much higher than those of *Chr4.2309.KCS* and *Chr4.2822.KCS*, and the expression of the latter two did not changed more at different seed development stages (Figure S18).

The proportion of NA in seed oil increased from 0% at 85 DAF to 1.96% at 100 DAF (Figure 4(c)), suggesting that this period marks the critical time for NA biosynthesis. We therefore compared these two time points, and found that the expression levels of *Chr4.2307.KCS*, *Chr4.2308.KCS*, and *Chr4.2311.KCS* were significantly upregulated (4.74‐, 4.25‐, and 3.28‐fold, respectively; *P* < 0.01) between 85 and 100 DAF (Table S25). These results were consistent with the RT‐PCR analysis at six seed development stages. The expression levels of *Chr4.2307.KCS*, *Chr4.2308.KCS*, ad *Chr4.2311.KCS* were indeed significantly upregulated from 85 DAF to 100 DAF (Figure S18). Furthermore, they were more highly expressed in the mature seeds than in the other plant tissues examined (root, stem, leaf, and flower) (Figure S19). These results suggested that these three genes are critically important in lipid metabolism and that they might be mainly responsible for regulating NA biosynthesis in *A. truncatum*.

A weighted gene co‐expression network analysis (WGCNA) was used to search for candidate genes associated with NA biosynthesis regulators. A total of 23 773 genes that are expressed among lines during seed development were chosen as probes for the WGCNA. The genes in one module (black module) were positively related to NA biosynthesis (*P* < 0.09) (Figure S20). The well‐known MYB and bZIP TFs were found to be involved in regulating NA biosynthesis (Table S26). Previous studies showed that MYB and bZIP TFs participate in regulating the synthesis of fatty acids (Wang et al., 2007; Yamamoto *et al*., 2009), and in particular, MYB had been found to play important roles in VLCFA biosynthesis (Raffaele *et al*., 2008).

## DISCUSSION

Aceraceae species are important ornamental foliage plants (Yang and Liu, 1998) that are distributed worldwide. High‐quality genome sequencing represents a key research option for studying specialized metabolites and the evolution of their biosynthesis (Afendi *et al*., 2012). *A. truncatum* is a versatile woody oil tree crop that produces NA. However, to date, the complete genome sequence of *A. truncatum* has not been reported. A sequence assembly and high‐quality sequencing based on PacBio RS II sequencing data combined with 10 × Genomics data represents a viable alternative to conventional genome sequencing. In this study, the *A. truncatum* genome was assembled into 13 pseudochromosomes based on Hi‐C technology, ultimately resulting in a high‐quality reference genome sequence of 633.28 Mb. The integrated strategy described herein was highly effective for the assembly of the complex *A. truncatum* genome.

### The evolutionary history of the *A. truncatum* genome

The characterization and annotation of the *A. truncatum* genome and a comparison with other plant genomes generated new information relevant for future investigations of the evolutionary history of *A. truncatum*. We updated the evolution of *A. truncatum* in Aceraceae species. In previous studies, Chen *et al*. (2019) and Ma *et al*. (2019) proved that *A. truncatum* and *A. miaotaiense* are closely related. In our study, we found that *A. truncatum* was not only closely related to *A. miaotaiense*, but also to *A. catalpifolium*, based on phylogeny of the chloroplast genomes. Up to now, the *A. yangbiense* genome was the only reported genome among Aceraceae species, so we compared the *A. truncatum* and *A. yangbiense* genomes. The phylogenetic analysis revealed they diverged about 9.4 Mya. WGDs are important for plant genome evolution (Panchy *et al*., 2016). We also determined that an ancient duplication occurred in *A. truncatum*, similar to most plant species (Jiao *et al*., 2011). There were no recent WGDs, with the exception of the γ event shared by all core eudicots (Jiao *et al*., 2011; Mcgrath *et al*., 2014), which is consistent with the results of the earlier evolution of Sapindales species, such as *A. yangbiense* (Yang *et al*., 2019), *C. sinensis* (Xu *et al*., 2013) and *Dimocarpus longan* (Lin *et al*., 2017).

### The *A. truncatum* genome contains a large number of repeat sequences

Repeat sequences are major components of eukaryotic genomes, and their activation might have caused the duplication and insertion events, leading to an increase in genome size (Levin and Moran, 2011; Kajitani *et al*., 2014). A previous study reported that *C. sinensis* (sweet orange) has a relatively compact genome among Sapindales species, in which the TEs account for 20.5% (Xu *et al*., 2013). We found that the number of TEs in *A. truncatum* is about threefold higher than in *C. sinensis*. Similar to the *A. truncatum* genome, the repeat sequences of most reported genomes in Sapindales species accounted for relatively high proportions, for example, 68.0% in *A. yangbiense* (Yang *et al*., 2019), 52.87% in *D. longan* (Lin *et al*., 2017), and 56.39 in *Xanthoceras sorbifolium* (Liang *et al*., 2019). Most plant genomes appear to contain abundant LTR retrotransposons. Similar to other genomes, LTRs were also dominant in the *A. truncatum* genome, especially *Copia* and *Gypsy*. These results will lay important foundations for studying the genome evolution of *Acer* species.

### The *A. truncatum* genome benefits NA research

Previous research reported that NA can be helpful in the treatment of brain diseases (Pamplona *et al*., 2005; Tanaka *et al*., 2007; Amminger *et al*., 2012). The NA biosynthesis pathway has been studied and some key components and enzyme‐catalyzed steps have been characterized (Taylor *et al*., 2009; Guo *et al*., 2009). Nevertheless, the mechanism underlying NA biosynthesis needs to be more thoroughly elucidated. In previous studies, *L. annua* and *C. graeca KCS* genes were cloned and heterologously expressed, resulting in an increase in the NA content (Guo *et al*., 2009; Taylor *et al*., 2009). However, the NA content was not significantly different between plants heterologously expressing *KCS* alone, *KCS* with *KCR*, *KCS* with *HCD*, *KCS* with *ECR*, or *KCS* with *KCR* and *HCD* (Dongxin *et al*., 2015), suggesting that *KCS* alone is crucial for NA biosynthesis. However, in the present study we detected no genes that were similar to the abovementioned cloned and heterologously expressed *KCS* genes (Guo, *et al*., 2009; Taylor *et al*., 2009).

Our further genomic analysis of paralogous *KCS* genes revealed that these genes are usually located within syntenic genomic blocks, which are the result of WGDs or segmental chromosomal duplications (Guo *et al*., 2016). A survey of tandem‐arrayed *KCS* genes was performed; the results showed that 10 of 28 *KCS* genes exhibited tandem arrays in *A. truncatum*. Interestingly, 10 and 6 *KCS* genes in *A. yangbiense* were clustered into a big branch. They are closely related to three *A. thaliana KCS* genes, namely, *KCS11*, *KCS2*, and *KCS20*, the latter two associated with cuticular wax biosynthesis (Saet‐Buyl *et al*., 2010). Although the *KCS* paralogous gene family expanded to 28 members, the evolution of the *KCS* gene family is essentially a history of gene duplications. The variations of different members resulted in the generation of product specificity and the emergence of completely new functions involved in other physiological processes (Vavouri *et al*., 2008; Guo *et al*., 2016). In our study, we found that only three of them (*Chr4.2307.KCS*, *Chr4.2308.KCS*, and *Chr4.2311.KCS*) were significantly upregulated at the critical time periods (from nothing to something) for NA biosynthesis. Moreover, their extremely high expression in seeds also indicated that these three *KCS* genes play an important role in seed development or fatty acid synthesis. Although family members share significant protein similarity, each may have partially overlapping or even distinct biological functions (Danilevskaya *et al*., 2007). The expansion and retention of these genes might facilitate natural selection of different catalytic functions and end products. Therefore, we speculate that the key genes of *A. truncatum* and *M. oleifera* play important roles in NA biosynthesis, which differ from those of *L. annua* and *C. graeca*.

A comparative transcriptome analysis, a WGCNA, and an examination of NA accumulation in developing seeds were combined to clarify which *KCS* genes contribute to NA biosynthesis. Finally, three *KCS* genes and related TFs were identified that might contribute to the regulation of NA biosynthesis. However, future transgenic studies will be needed to confirm the roles of the *KCS* genes in NA biosynthesis in *A. truncatum*, to elucidate whether a major single gene or the cooperation of three *KCS* genes is centrally important.

In summary, our study provides critical information regarding Aceraceae genomes, which has been limited to date. The elucidation of the *A. truncatum* genome sequence described herein and the sequence details deposited in the NCBI database will benefit future research on *A. truncatum* flowering, seed production, and resistance to biotic and abiotic stresses. Therefore, our findings can be expected to contribute to the further characterization of *A. truncatum* as an economically important tree species that produces NA in its seed oil.

## EXPERIMENTAL PROCEDURES

### Plant materials

The diploid monoecious *A. truncatum*, originally collected in Nanjing, Jiangsu Province, was preserved in the Jiangsu Province Aceraceae Germplasm Repository (Lishui, Nanjing). The materials used for genome sequencing and assembly were healthy and young leaves.

### DNA extraction and sequencing

Genomic DNA was extracted from leaves of *A. truncatu*m using the DNAsecure Plant Kit (TIANGEN, Biotech Co., Ltd., Beijing, China). Sequencing libraries with an insert size of 350 bp were constructed using a library construction kit (Illumina, CA, USA). These libraries were then sequenced using an Illumina HiSeq X10 platform. Raw reads were filtered according to sequencing quality, the presence of adapter contamination, and duplication. Only high‐quality reads were used for genome assembly. For a PacBio 20‐kb insert size library, at least 10 μg of sheared DNA was required. SMRTbell template preparation involved DNA concentration, damage repair, end repair, ligation of hairpin adapters, and template purification. Finally, we carried out 20‐kb single‐molecule real‐time DNA sequencing by PacBio and sequenced the DNA library on the Pacbio Sequel platform. DNA sample preparation, indexing, and barcoding were done using the GemCode Instrument from 10x Genomics. About 1 ng input DNA was used for the GEM reaction procedure during PCR, and 16‐bp barcodes were introduced into droplets. Then, the droplets were fractured following the purification of the intermediate DNA library. 10x Genomics libraries were finally sequenced on the Illumina Hiseq X10.

For the Hi‐C library, chromatin was fixed in place with formaldehyde in the nucleus. Fixed chromatin was digested, 5′ overhangs were filled in with biotinylated nucleotides, and free blunt ends were ligated. After ligation, cross‐links were reversed and the DNA was purified from protein. Purified DNA was treated to remove biotin that was not internal to the ligated fragments. The DNA was then sheared to an average fragment size of ~350 bp, and sequencing libraries were generated using NEBNext Ultra enzymes and Illumina‐compatible adapters. Biotin‐containing fragments were isolated using streptavidin beads before PCR enrichment of each library. The libraries were sequenced on an Illumina Novaseq platform.

### Genome assembly

The long subreads generated by the PacBio platform were assembled by the following procedures: The initial assembly was first generated by Falcon (Chin *et al*., 2016) using pre‐assemble reads after error correction (up to 99.999%). After the initial assembly, FALCON‐Unzip (Chin *et al*., 2016) was used to produce primary contigs (p‐contigs), which were polished using Quiver (https://github.com/PacificBiosciences/GenomicConsensus). Lastly, Pilon (Walker *et al*., 2014) was used to perform error correction of p‐contigs with the short paired‐end reads and high‐quality consensus sequences were obtained. Finally, scaffolding was performed by FragScaff (Mostovoy *et al*., 2016) with the barcoded sequencing reads generated by 10x Genomics.

To obtain chromosome‐level assembly, the Hi‐C clean data were aligned to the preceding assembly using BWA software (Li and Durbin, 2009). Only the read pairs with both reads aligned to contigs were considered for scaffolding. For each read pair, its physical coverage was defined as the total bases spanned by the sequence of reads and the gap between the two reads when mapped to contigs. Per‐base physical coverage for each base in the contig was defined as the number of read pairs’ physical coverage. Mis‐assembly could be detected by the sudden drop in per‐base physical coverage in a contig. According to physical coverage of the alignment result, the mis‐assemblies would be sheared to correct the mis‐assemble errors by SALSA (Jay *et al*., 2017). According to the linkage information and restriction enzyme site, the string graph formulation was used to construct the scaffold graph with LACHESIS (Burton *et al*., 2013).

To assess the accuracy and completeness of the assemblies, the Illumina clean reads were mapped to the corresponding assembly using BWA (Li and Durbin, 2009). CEGMA (http://korflab.ucdavis.edu/dataseda/cegma/) (Parra *et al*., 2007) and BUSCO (http://busco.ezlab.org/) (Simao *et al*., 2015) were also used to assess the completeness of our assemblies. The assembly quality of gene‐coding regions was evaluated by aligning the transcriptome sequenced from whole‐plant RNAs using BLAT (Kent, 2002). In addition, the LAI score (Ou *et al*., 2018), a standard for evaluating the assembly of repeat sequences, was used to evaluate the assembly continuity.

### Repetitive elements annotation

A combined strategy based on sequence homology and *de novo* prediction was employed to identify the repetitive elements of our assemblies. Tandem repeats were also identified by the software Tandem Repeats Finder (http://tandem.bu.edu/trf/trf.html). For the *de novo* search, we searched repeat elements by RepeatModeler (http://www.repeatmasker.org/RepeatModeler.html), RepeatScout (Price *et al*., 2005), Piler (Edgar and Myers, 2005), and LTR‐Finder (Xu and Wang, 2007), with default parameters. The non‐redundancy repeat library was finally put into Repeatmasker (http://www.repeatmasker.org) to predict repeat elements. For the homology‐based prediction, RepeatProteinMask (http://www.repeatmasker.org) was used to identify repeats compared with the known consensus sequences of the Repbase library (Jurka *et al*., 2005).

To identify complete LTR‐retrotransposons, LTRfinder (Xu and Wang, 2007) (parameters: ‐l 1000 ‐L 20 000 ‐d 100 ‐D 5000 ‐M 0.3) and LTRharvest (Ellinghaus, *et al*., 2008) (parameters: ‐v ‐mintsd 4 ‐maxtsd 6) were used to identify the candidate LTR‐retrotransposons. Then LTRdigest (Steinbiss *et al*., 2009) was used to identify candidate retrotransposons by searching for known protein domains (parameters: ‐trnas ‐hmms). Elements containing GAG domains, protease domains, reverse transcriptase domains, and integrase domains were considered as intact. The predicted LTRs were extracted and aligned with muscle (Edgar, 2004), and the distance K was calculated with the Jukes–Cantor model by an in‐house Perl script and the insert time of each LTR‐retrotransposon was calculated by the following formula: *T* = *K*/(2 × *r*), where *r* refers to a general substitution rate of 1.3 × 10^−8^ per site per year.

### Centromere region identification

The centromeric regions of *A. truncatum* were identified using an approach described by VanBuren *et al*. (2015). Tandem Repeat Finder (Benson, 1999) was used to identify all tandem repeats. The base centromere repeats were identified by trf_cluster (downloaded from http://korflab.ucdavis.edu/datasets/Centromere_data/). To determine the location of centromeres, the base centromere repeat was aligned to the genome using BLASTN (E‐value < 1E−5, overlap > 90%). The above approaches were applied to detect the centromere regions of *A. yangbiense*.

### Gene prediction and annotation

To predict genes in the *A. truncatu*m genome, we performed a combination of *de novo*, homology, and transcript‐based approaches. For the homology‐based prediction, the protein sequences of *A. thaliana* (TAIR10), *C. sinensis* (Xu *et al*., 2013), *Glycine max* (V1.0), *G. raimondii* (GCF_000327365), *Populus trichocarpa* (JGI2.0), *S. lycopersicum* (SL2.50), and *Oryza sativa* (IRGSP‐1.0) were downloaded from the NCBI, Ensemble, and JGI databases, and aligned to the *A. truncatu*m genome by TBLASTN with E‐value < 1E−5. GeneWise (version 2.4.1) (Birney *et al*., 2004) was used to annotate genes with the alignments. The clean RNA‐seq data (root, stem, leaf, flower, and mature seed) were aligned to the genome by Tophat (version 2.0.13) (Trapnell *et al*., 2009) and assembled into gene models by Cufflinks (version 2.1.1) (http://cole‐trapnell‐lab.github.io/cufflinks/releases/v2.1.1/). Besides, we applied Trinity (version 2.0) (Grabherr *et al*., 2011) to assemble the RNA‐seq data, and then pasa software (Haas *et al*., 2003) was used to improve the gene structures. For the *de novo* prediction, we annotated genes by augustus (version 2.5.5) (Stanke and Morgenstern, 2005), GlimmHMM (version 3.0.1) (Majoros *et al*., 2004), SNAP (Korf, 2004), GeneScan (version 1.0) (Aggarwal and Ramaswamy, 2002), and GeneID (v1.4) (Parra, *et al*., 2000). The gene models retrieved from all methods were integrated by EvidenceModeler (Haas *et al*., 2008). The software pasa (Haas *et al*., 2003) was then used to obtain the information of genes’ untranslated regions and alternative splicing variations. We applied InterProScan (Zdobnov and Apweiler, 2001) to determine the domains and motifs of the gene set. GO IDs were derived from the InterPro entry. Gene functions were obtained according to the best match of the search against the NCBI NR, Swiss‐Prot, and KEGG databases, using BLASTP (E‐value < 1E−5).

### Genome comparison and evolution

We performed a comparative genome analysis to identify gene family clusters among *A. truncatu*m, *A. yangbiense* (Yang *et al*., 2019), *O. sativa* (IRGSP‐1.0), *A. thaliana* (TAIR10), *C. sinensis* (Xu *et al*., 2013), *G. raimondii* (GCF_000327365)*, Juglans regia* (GCF_001411555.1_wgs.5d), *Glycine max* (v1.0), *Vitis vinifera* (IGGP_12x), *Olea europae* (GCF_002742605.1_O_europaea_v1), *Sesamum indicum* (GCF_000512975.1_S_indicum_v1.0), *Helianthus annuus* (HanXRQr1.0), *P. trichocarpa* (JGI2.0), *S. lycopersicum* (SL2.50), and *M. Oleifera* (Xu *et al*., 2019). The longest protein sequences (longer than 50 amino acids) of all species were searched against each other using BLASTP (E‐value < 1E−7) (https://blast.ncbi.nlm.nih.gov/Blast.cgi) and clustered using the OrthoFinder algorithm (inflation parameter: 1.5) (version 2.2.6) (Emms and Kelly, 2019). A dataset of single‐copy orthologous genes (only one gene copy per species in the cluster) was then used for muscle (Edgar, 2004) alignment, and the phylogenetic tree was constructed using RAxML (Stamatakis, 2014). Mcmctree in PAML packages (Yang, 1997) was performed to estimate the divergence time, with four corrected divergence time points from the TimeTree website (http://www.timetree.org/): Monocots versus Dicots (173–148 Mya), *A. thaliana* versus *P. trichocarpa* (109–97 Mya), *A. thaliana* versus *V. vinifera* (115–105 Mya), and *H. annuus* L. versus *S. lycopersicum* (107–93 Mya). café (De et al., 2006) was used to analyze the expansion/contraction of gene families. In addition, the chloroplast sequences of 17 Aceraceae species and *D. longan* were downloaded from the NCBI database and clustered using OrthoFinder (inflation parameter: 1.5) (version 2.2.6) (Emms and Kelly, 2019). A dataset of single‐copy orthologous genes (only one gene copy per species in the cluster) was then used for muscle (Edgar, 2004) alignment, and the phylogenetic tree was constructed using RAxML (Stamatakis, 2014).

### The whole‐genome duplication and synteny analysis

Syntenic blocks in the *A. truncatum* genome were detected using MCScanX (http://chibba.pgml.uga.edu/mcscan2) (Wang *et al*., 2012), after an all‐to‐all BLASTP (https://blast.ncbi.nlm.nih.gov/Blast.cgi) search for the best hit. To estimate WGD events, we calculated 4DTv values of paralogous genes of *A. truncatum* using in‐house Perl scripts. 4DTv was the return value of 4DT (third codon transversions within these fourfold degenerate sites) divided by 4D (the fourfold degenerate sites) and corrected by the HKY substitution model. The syntenic blocks within *A. yangbiense*, *C. sinensis*, *P. trichocarpa*, and *M. oleifera* were identified and 4DTv values were calculated by the same method. The syntenic regions between *A. truncatum*, *A. yangbiense*, *P. trichocarpa*, and *C. sinensis* were also searched by MCScanX (Wang *et al*., 2012) and the 4DTv value of orthologous genes was calculated.

### Lipid analysis and oil body obesrvation

The 50 kernels from different developmental stages (70, 85, 100, 115, 145, and 180 DAF) were dried at 65°C for 72 h. The seed oil was extracted with petroleum ether (30–60°C) using a Soxhlet apparatus and the distillation temperature was kept at 65°C for 24 h. Then lipid was separated using a rotary vacuum evaporator at 65°C. To make fatty acid methyl esters (FAMEs), about 0.2 ml oil was dissolved in 2 ml of benzene/petroleumether (1:1, v/v), which was then mixed with 2 ml of KOH‐CH_3_OH (0.4 m) as described by Gao (2006). The oil was extracted, and the esterification reactions were directly performed in a rotary vacuum evaporator. The product was diluted to 10 ml with deionized water, and the upper phase was used to analyze fatty acid composition by gas chromatography‐mass spectrometry (GC/MS) using a TRACE DSQ GC/MS (Thermo Fisher Scientific Inc., Waltham, MA, USA). The procedures were performed as described by Zhang *et al*. (2018). The standards (Seebio Biotech Co., Ltd, Shanghai, China) were used to make standard curves and perform sample analysis. The amounts of fatty acids were calculated by comparing the peak areas of FAMEs with the standard (Kim *et al*., 2015). Transmission electron microscopy samples were cut from the middle section of each embryo (JEM‐1400; JEOL, Tokyo, Japan) and prepared following the procedure described by Chen *et al*. (2012). Brief, the samples were immediately fixed with 4% (v/v) glutaraldehyde in sodium phosphate buffer (pH 7.2), post‐fixed in osmium tetroxide, dehydrated, and embedded in Spurr’s resin. The sections were observed at 60 kV with an electron microscope (JEM‐1200; JEOL).

### Transcriptome library preparation and gene expression analysis

We collected *A. truncatu*m roots, stems, leaves, flowers, and developing seeds (70, 85, 100, 115, 145 and 180 DAF) from three plants (with three biological repeats each). Total RNA was extracted using TRIzol® Reagent (Thermo Fisher Scientific) according to the manufacturer’s instructions, and the RNA‐seq libraries were constructed using the NEBNext Ultra Directional RNA Library Prep Kit (NEB, USA). The RNA‐seq libraries were then sequenced on an Illumina Novaseq platform and 150‐bp paired‐end reads were generated. After the quality control (reads that containing adapters, reads containing poly‐N, and low‐quality reads were removed) by an in‐house Perl script, the clean reads were mapped to the genome using HISTA2 (version 2.0.4) (Kim *et al*., 2015). The Reads Per Kilobase per Million mapped reads (RPKM) values were used to analyze the expression level of each gene for further calculation of the differentially expressed genes using DESeq2 (https://bioconductor.org/packages/release/bioc/html/DESeq2.html).

The validation of differentially expressed genes was performed by quantitative RT‐PCR (qRT‐PCR). Total RNA was extracted as described above, and cDNA was reverse transcribed from 1 μg total RNA using M‐MLV reverse transcriptase (Promega, Madison, WI). The cDNA templates were diluted 20‐fold before use. qRT‐PCR was performed with in a Step One Plus Real‐Time PCR System (Applied Biosystems) using SYBR Premix ExTaq^TM^ (TaKaRa) according to the manufacturer’s protocol. The specific primers were designed with Primer5 (Tables S27 and S28). *Actin* was selected as the internal reference gene (Wang *et al*., 2018). The expression of each gene was analyzed in three biological replicates and three technical repetitions. Corresponding gene expression levels were analyzed with the 2^−∆∆Ct^ method.

### The identification and phylogenetic analyses of NA biosynthesis genes and *LEA* genes in *A. truncatu*m

We used different methods to identify NA biosynthesis genes and *LEA* genes. To identify homologous genes in NA biosynthesis pathways, the sequences of proteins from the *A. thaliana* fatty acid biosynthesis pathway were downloaded (http://aralip.plantbiology.msu.edu/) and used for BLASTP (https://blast.ncbi.nlm.nih.gov/Blast.cgi) queries. The genes were selected if the following requirements were met: (i) filtering for hits with E‐value ≤ 1E−5, alignment identity ≥ 50%, and alignment coverage ≥ 50% of the query genes; (ii) having the same Pfam domains as the query genes. These genes were also searched in *A. yangbiense*, *C. sinensis*, and *M. oleifera* by the same method. The Hidden Markov Model profiles of the LEA protein domains PF03760 (LEA1), PF03168 (LEA2), PF03242 (LEA3), PF02987 (LEA4), PF00477 (LEA5), PF00257 (DEHYDRIN), and PF04927 (SMP) were used to query the protein dataset of *A. truncatum* by HMMER (http://hmmer.janelia.org/) with E‐value < 0.01. Multiple sequence alignment of the amino acid sequences of NA biosynthesis genes and *LEA* genes were performed respectively using the default parameters of ClustalW, and then phylogenetic analyses were conducted by RAxML (Stamatakis, 2014).

### WGCNA (Weighted Gene Co‐Expression) network analysis and the identification of transcription factors

The datasets of the six seed development stages were averaged and filtered to remove non‐expressed genes (RPKM < 1). The co‐expression network was generated using the RPKM values of the filtered genes by the WGCNA package in R (Langfelder and Horvath, 2008; Team, 2016). To relate the physiology measurements with the network, the module eigengenes were correlated with the fatty acid contents data. TTFs within modules were identified and classified into different families using iTAK software (Zheng *et al*., 2016).

## CONFLICT OF INTEREST

The authors declare no conflict of interest.

## AUTHOR CONTRIBUTIONS

QYM, QZL, XZ, and SXJ designed the project and the strategy; SXL, JW, LZ, KYY, and SXL contributed to plant sample collection, DNA/RNA preparation, library construction, and sequencing; QYM, TLS, QZL, JFM, and XX worked on genome assembly and annotation and comparative analyses; QYM, TLS, SXL, JW, YNW, and LZ performed transcriptome and genetic analyses and identified candidate genes; QYM, TLS, TMY, QZL, XZ, and SXJ wrote and revised the manuscript.

## Supporting information


**Figure S1.** The K‐mer analysis of *A. truncatum*. The frequency of 17‐mers is exhibited by 17‐bp sequences within filtered reads of the 350‐bp inset size library. As the curve shows, the peak depth is 76, and the genome size was estimated as 662.08 Mb (653.44 Mb after revision).
**Figure S2.** The Hi‐C chromatin interaction map of 13 pseudomolecules in *A. truncatum*.
**Figure S3.** The insert time of LTR retrotransposons in *A. truncatum*, *A. yangbiense, C. sinensis*, and *M. oleifera*. The red, green, and blue regions represent the LTR insertion time (million years ago) of Atru: *A. truncatum*, Ayan: *A. yangbiense*; Csin: *C. sinensis*, and Mole: *M. oleifera*.
**Figure S4.** Gene numbers in each group defined by OrthoMCL.
**Figure S5.** The phylogeny and divergence time of *A. truncatum* and 14 other plants.
**Figure S6.** The phylogeny based on coding sequences of single‐copy orthologous genes shared among 17 chloroplast genomes in Aceraceae species. *Dimocarpus longan* served as the outgroup.
**Figure S7.** The phylogeny based on protein sequences of single‐copy orthologous genes shared among 17 chloroplast genomes in Aceraceae species. *Dimocarpus longan* served as the outgroup.
**Figure S8.** Synteny comparison of *A. truncatum* and *C. sinensis*. The outermost circle (blue) displays ideograms of the pseudochromosomes of the genomes. Atru: *A. truncatum*, Csin: *C. sinensis*. The second circle displays gene density. The innermost circle displays homologies between *A. truncatum* and *C. sinensis*. All distributions are drawn in a window size of 300 kb, chromosomes_units = 500 000.
**Figure S9.** Synteny comparison of *A. truncatum* and *A. yangbiense*. The outermost circle (blue) displays ideograms of the pseudochromosomes of the genomes. Atru: *A. truncatum,* Ayan: *A. yangbiense*. The second circle displays gene density. The innermost circle displays homologies between *A. truncatum* and *A. yangbiense*. All distributions are drawn in a window size of 300 kb, chromosomes_units = 500 000.
**Figure S10.** Syntenic pattern of *A. truncatum* with *A. yangbiense* and *C. sinensis*. The *x*‐axis indicates that each gene of one genome has no orthologous region, one orthologous region, two orthologous regions, three orthologous regions, or four orthologous regions with another genome. The *y*‐axis indicates the percentage of the gene blocks in genomes.
**Figure S11.** The phylogeny analysis of *LEA* genes in *A. truncatum*.
**Figure S12.** The distribution of *LEA* genes in *A. truncatum*.
**Figure S13.** Heat map of gene expression profiles of *LEA* genes. (a) Differentially expressed *LEA* genes during seed development. (b) Differentially expressed *LEA* genes in five tissues.
**Figure S14.** Quantitative PCR of *LEA* genes in different tissues. Error bars indicate standard errors of the mean from three technical replicates.
**Figure S15.** Quantitative PCR of *LEA* genes in six seed development stages. Error bars indicate standard errors of the mean from three technical replicates.
**Figure S16.** The phylogenetic tree and distribution of *KCS* genes based on coding sequences of *A. truncatum*. (a) The maximum likelihood tree of *KCS* genes from *A. truncatum* (red triangle), *A. yangbiense* (write triangle), *M. oleifera* (blue square), and *A. thaliana* (write circle).
**Figure S17.** The expression profiles of *KCS* genes in different tissues of *A. truncatum*. *Z*‐scores were calculated after log_10_ transformation of FPKM values plus 1.
**Figure S18.** Quantitative PCR of *KCS* genes in six seed development stages. Error bars indicate standard errors of the mean from three technical replicates.
**Figure S19.** Quantitative PCR of *KCS* genes in different tissues. Error bars indicate standard errors of the mean from three technical replicates.
**Figure S20.** WGCNA of fatty acid contents with RNA‐seq data of seed development stages in *A. truncatum*. The numbers within the heat map represent correlations (red, positively correlated; blue, negatively correlated) and *P*‐values (in parentheses) for the module–trait associations.

**Table S1.** K‐mer statistics of *A. truncatum*.
**Table S2.** Sequencing data of *A. truncatum*.
**Table S3.** Summary of *A. truncatum* pseudomolecules.
**Table S4.** The Hi‐C assembly results of *A. truncatum*.
**Table S5.** Coverage statistics of the *A. truncatum* genome.
**Table S6.** CEGMA evaluation results.
**Table S7.** BUSCO notation assessment of *A. truncatum* genome and annotation.
**Table S8.** Assessing the gene region assembly by mapping the RNA assembly to the *A. truncatum* genome.
**Table S9.** Statistical results of gene functional annotations.
**Table S10.** Statistical results of non‐coding RNAs.
**Table S11.** Classification of repetitive elements in the *A. truncatum* genome.
**Table S12.** The LTR subfamilies in *A. truncatum* and other published genomes.
**Table S13.** The seed sequences of centromeric regions of *A. truncatum* and *A. yangbiense* chromosomes.
**Table S14.** Details of the chloroplast genome sequences used for the phylogenetic analysis.
**Table S15.** GO enrichment results for expanded genes in *A. truncatum*.
**Table S16.** GO enrichment results for contracted genes in *A. truncatum*.
**Table S17.** GO enrichment results for the genes in *A. truncatum*‐specific gene families.
**Table S18.** KEGG enrichment results for expanded genes in *A. truncatum*.
**Table S19.** GO enrichment results for expanded genes in *A. yangbiense*.
**Table S20.** GO enrichment results for contracted genes in *A. yangbiense*.
**Table S21.** KEGG enrichment results for expanded genes in *A. yangbiense*.
**Table S22.** A summary of drought‐tolerant genes identified in *A. truncatum* and other published genomes.
**Table S23.** A summary of drought tolerance‐related transcription factors identified in *A. truncatum* and other published genomes.
**Table S24.** The copy numbers of VLCFA biosynthesis‐related genes in 12 plants.
**Table S25.** Expression levels of VLCFA biosynthesis‐related genes that are significantly upregulated between 85 DAF and 100 DAF and are involved in fatty acid elongation.
**Table S26.** The transcription factors (TFs)/transcription regulatory factors (TRs) in the black module of WGCNA analysis.
**Table S27.** List of *KCS* gene‐specific primers (5’–3’) used for RT‐PCR.
**Table S28.** List of *LEA* gene‐specific primers (5’–3’) used for RT‐PCR.

## Data Availability

The *A. truncatum* genome has been deposited under BioProject accession number PRJNA557096 and BioSample accession number SAMN12389479. PacBio and Illumina reads, resequencing sequences reads and Hi‐C data have been submitted in the Sequence Read Archive (SRA) under study accession number SUB6287730. The final assembled version(s) must be available in https://doi.org/10.6084/m9.figshare.12986237.v2.
