# Supplementary material for: The Acer truncatum genome provides insights into nervonic acid biosynthesis
Source: Plant J. 2020 Oct 2;104(3):662–78. doi: 10.1111/tpj.14954 (PMC7702125; doi:10.1111/tpj.14954)
Supplement: Supplementary file 1 — Figure S1. The K‐mer analysis of A. truncatum. The frequency of 17‐mers is exhibited by 17‐bp sequences within filtered reads of the 350‐bp inset size library. As the curve shows, the peak depth is 76, and the genome size was estimated as 662.08 Mb (653.44 Mb after revision). Figure S2. The Hi‐C chromatin interaction map of 13 pseudomolecules in A. truncatum. Figure S3. The insert time of LTR retrotransposons in A. truncatum, A. yangbiense, C. sinensis, and M. oleifera. The red, green, and blue regions represent the LTR insertion time (million years ago) of Atru: A. truncatum, Ayan: A. yangbiense; Csin: C. sinensis, and Mole: M. oleifera. Figure S4. Gene numbers in each group defined by OrthoMCL. Figure S5. The phylogeny and divergence time of A. truncatum and 14 other plants. Figure S6. The phylogeny based on coding sequences of single‐copy orthologous genes shared among 17 chloroplast genomes in Aceraceae species. Dimocarpus longan served as the outgroup. Figure S7. The phylogeny based on protein sequences of single‐copy orthologous genes shared among 17 chloroplast genomes in Aceraceae species. Dimocarpus longan served as the outgroup. Figure S8. Synteny comparison of A. truncatum and C. sinensis. The outermost circle (blue) displays ideograms of the pseudochromosomes of the genomes. Atru: A. truncatum, Csin: C. sinensis. The second circle displays gene density. The innermost circle displays homologies between A. truncatum and C. sinensis. All distributions are drawn in a window size of 300 kb, chromosomes_units = 500 000. Figure S9. Synteny comparison of A. truncatum and A. yangbiense. The outermost circle (blue) displays ideograms of the pseudochromosomes of the genomes. Atru: A. truncatum, Ayan: A. yangbiense. The second circle displays gene density. The innermost circle displays homologies between A. truncatum and A. yangbiense. All distributions are drawn in a window size of 300 kb, chromosomes_units = 500 000. Figure S10. Syntenic pattern of A. truncatum wi [file TPJ-104-662-s001.pdf]

The *Acer truncatum* genome provides insights into the nervonic acid biosynthesis

Supplementary information

This document includes the following:
- 20 supplementary figures

Supplementary Figure Legends

Figure S1. The K-mer analysis of *A. truncatum*. The frequency of 17-mers are exhibited by 17 bp sequences within filtered reads of 350bp inset size library. As the curve shows, the peak depth is 76, and the genome size was estimated as 662.08Mb (653.44Mb after revised).

Figure S2. The Hi-C chromatin interaction map of 13 pseudomolecules in *A. truncatum*.

Figure S3. The insert time of LTR retrotransposons in *A. truncatum*, *A. yangbiense, C. sinensis* and *M. oleifera*.The red, green and blue region represent the LTR insertion time (million years ago) of Atru: *A. truncatum*, Ayan: *A. yangbiense*; Csin*: C. sinensis* and Mole:*M. oleifera*, respectively.

Figure S4. Gene numbers in each group defined by OrthoMCL.

Figure S5. The phylogeny and divergence time of *A. truncatum* and 14 other plants.

Figure S6. The phylogeny based on coding sequence of single-copy orthologous gene shared among 17 chloroplast genomes in Aceraceae species. *Dimocarpus longan* served as the outgroup.

Figure S7. The phylogeny based on protein sequences of single-copy orthologous gene shared among 17 chloroplast genomes in Aceraceae species. *Dimocarpus longan* served as the outgroup.

Figure S8. Synteny comparison of *A. truncatum* and *C. sinensis*. The outmost circle (blue) displays ideograms of the pseudochromosomes of the genomes. Atru: *A. truncatum*, Csin: *C. sinensis.* The second circle displays gene density. The innermost circle displays homologies between *A. truncatum* and *C. sinensis.* All distributions are drawn in a window size of 300 kb,

chromosomes_units = 500000.

Figure S9. Synteny comparison of *A. truncatum* and *A. yangbiense*. The outmost circle (blue) displays ideograms of the pseudochromosomes of the genomes. Atru: *A. truncatum,* Ayan: *A. yangbiense.* The second circle displays gene density. The innermost circle displays homologies between *A. truncatum* and *A. yangbiense.* All distributions are drawn in a window size of 300 kb, chromosomes_units = 500000.

Figure S10. Syntenic pattern of *A. truncatum* with *A. yangbiense* and *C. sinensis*. The x-axis represents that each gene of one genome has no orthologous region, one orthologous region, two orthologous regions, three orthologous regions and four orthologous regions with another genome. The y-axis is the percentage of the gene blocks in genomes.

Figure S11. The phylogeny analysis of *LEA* genes in *A. truncatum*.

Figure S12. The distribution of *LEA* genes in *A. truncatum*.

Figure S13. Heat map of gene expression profiles of *LEA* genes. (a) Differential expression *LEA* genes during seed development times; (b) Differential expression *LEA* genes in five tissue.

Figure S14. Quantitative PCR of *LEA* genes in different tissues. Error bars are standard errors of the mean from three technical replicates.

Figure S15. Quantitative PCR of *LEA* genes in six seed-development stages. Error bars are standard errors of the mean from three technical replicates.

Figure S16. The phylogenetic tree and distribution of *KCS* genes based on coding sequences of *A. truncatum*. The phylogenetic tree and distribution of *KCS* genes. (a)The ML tree of *KCS* genes. *A. truncatum* (red triangle), *A. yangbiense* (write triangle), *M. oleifera* (blue square), *A. thaliana* (write circle).

Figure S17. The expression profiles of *KCS* genes in different tissues of *A. truncatum*. Z-scores were calculated after log10 transformation of FPKM values plus 1.

Figure S18. Quantitative PCR of *KCS* genes in six seed-development stages. Error bars are standard errors of the mean from three technical replicates.

Figure S19. Quantitative PCR of *KCS* genes in different tissues. Error bars are standard errors of the mean from three technical replicates.

Figure S20. WGCNA analysis of fatty acid contents with RNA-seq data of seed development stages in *A. truncatum*. The numbers within the heat map represent correlations (red, positively

correlated, blue, negatively correlated) and P-values (in parentheses) for the module-trait associations.

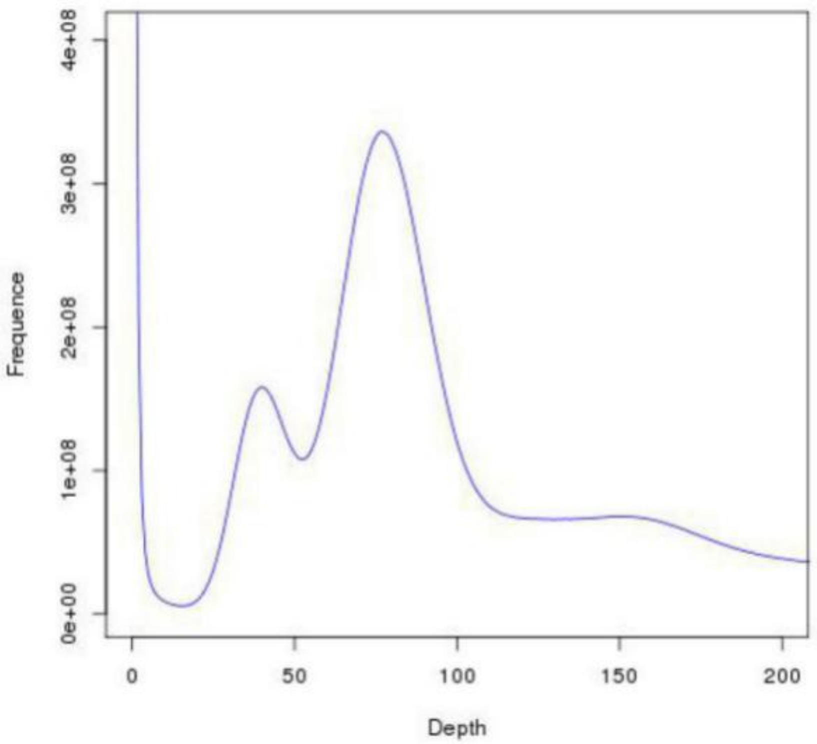

Figure S1. The K-mer analysis of *A. truncatum*. The frequency of 17-mers are exhibited by 17 bp sequences within filtered reads of 350bp inset size library. As the curve shows, the peak depth is 76, and the genome size was estimated as 662.08Mb (653.44Mb after revised).

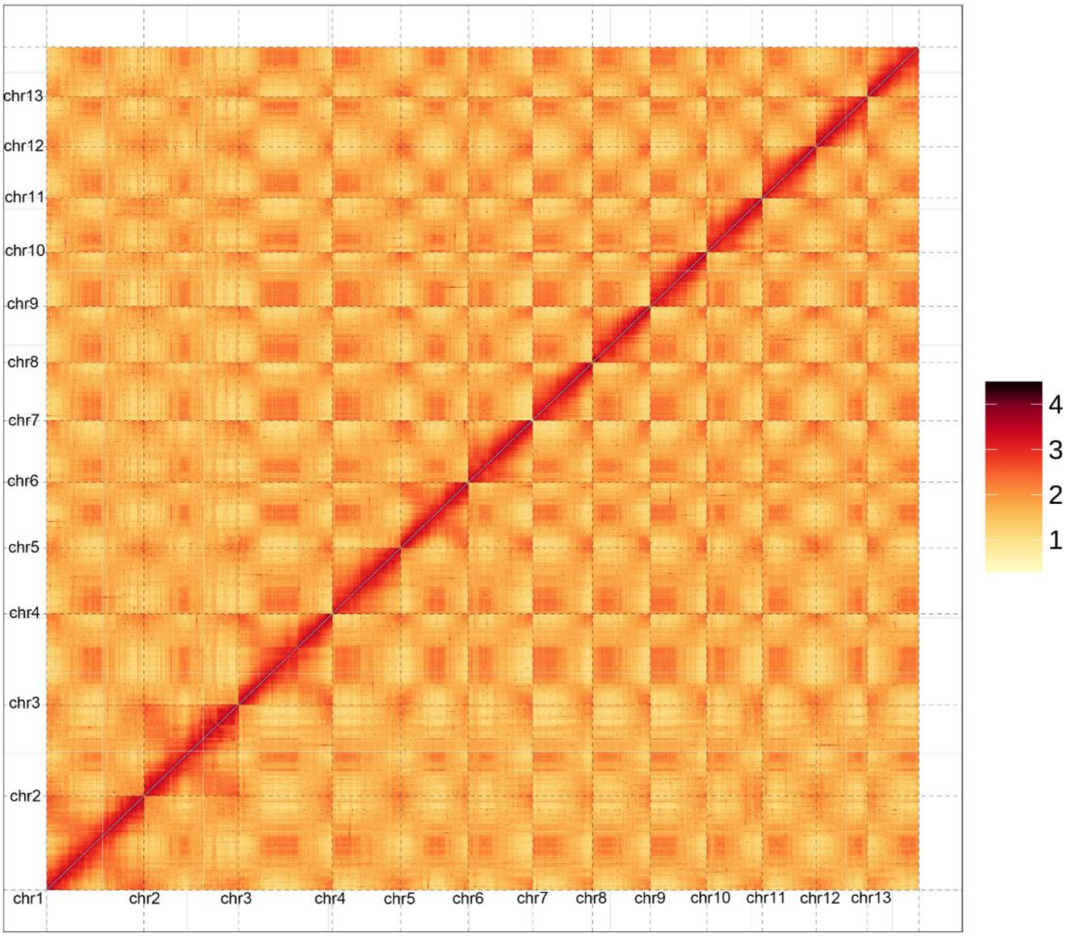

Figure S2. The Hi-C chromatin interaction map of 13 pseudomolecules in *A. truncatum*.

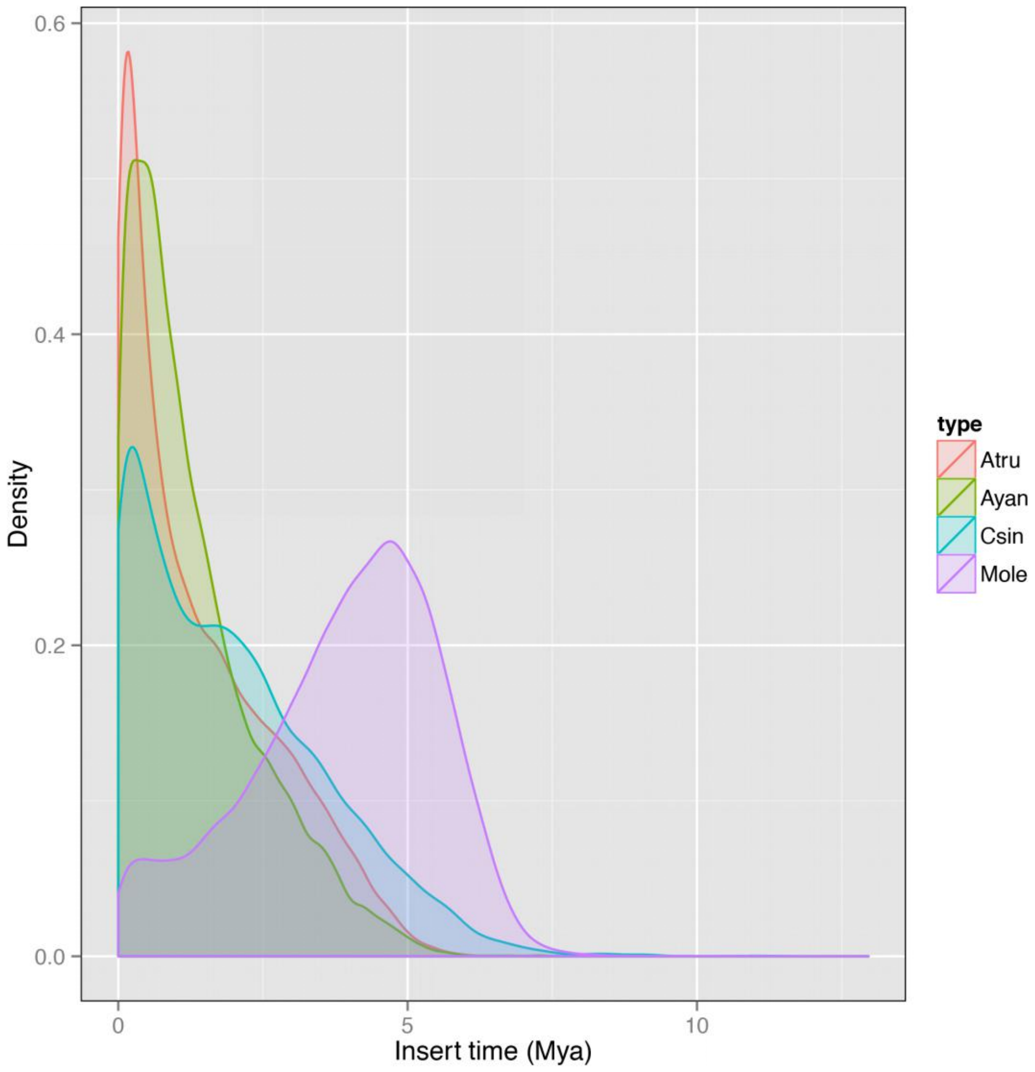

Figure S3. The insert time of LTR retrotransposons in *A. truncatum*, *A. yangbiense, C. sinensis* and *M. oleifera*. The x axis is the period (Mya, that is million years ago) that LTR inserted into the genome of Atru: *A. truncatum* (red), Ayan: *A. yangbiense* (green); Csin*: C. sinensis* (blue) and Mole: *M. oleifera* (purple).The y axis is the density of those LTR retrotransposons.

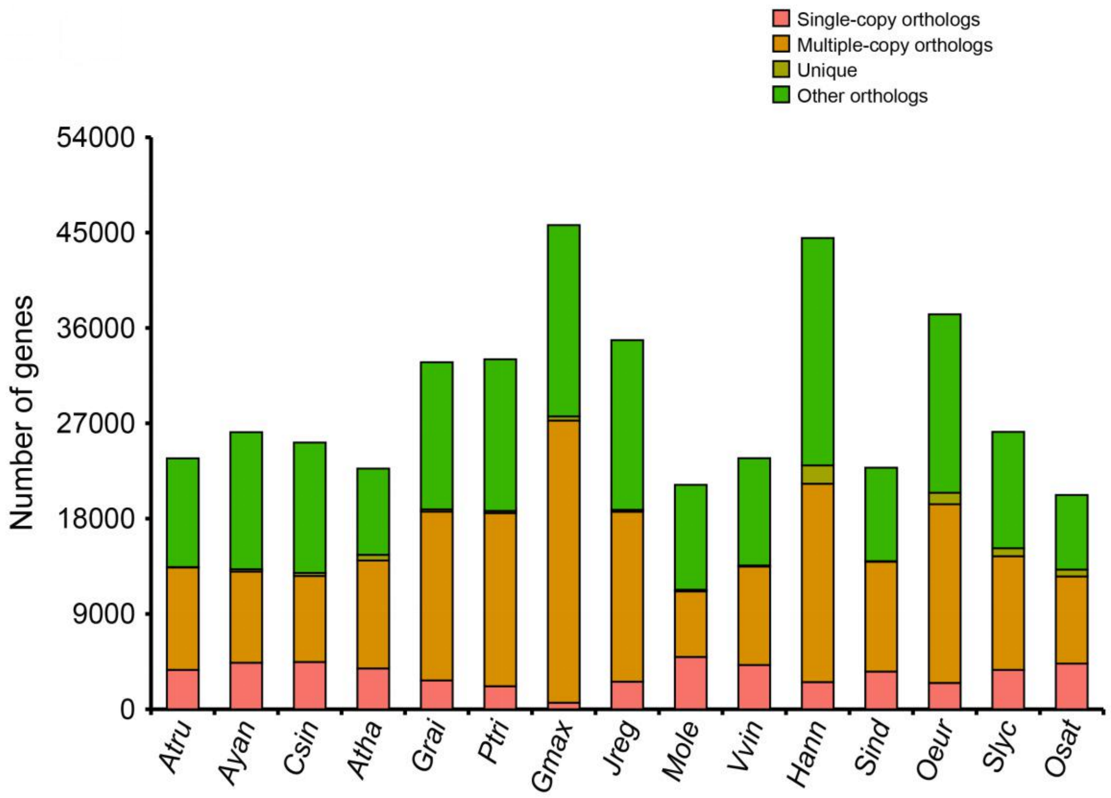

Figure S4. Gene numbers in each group defined by OrthoFinder.

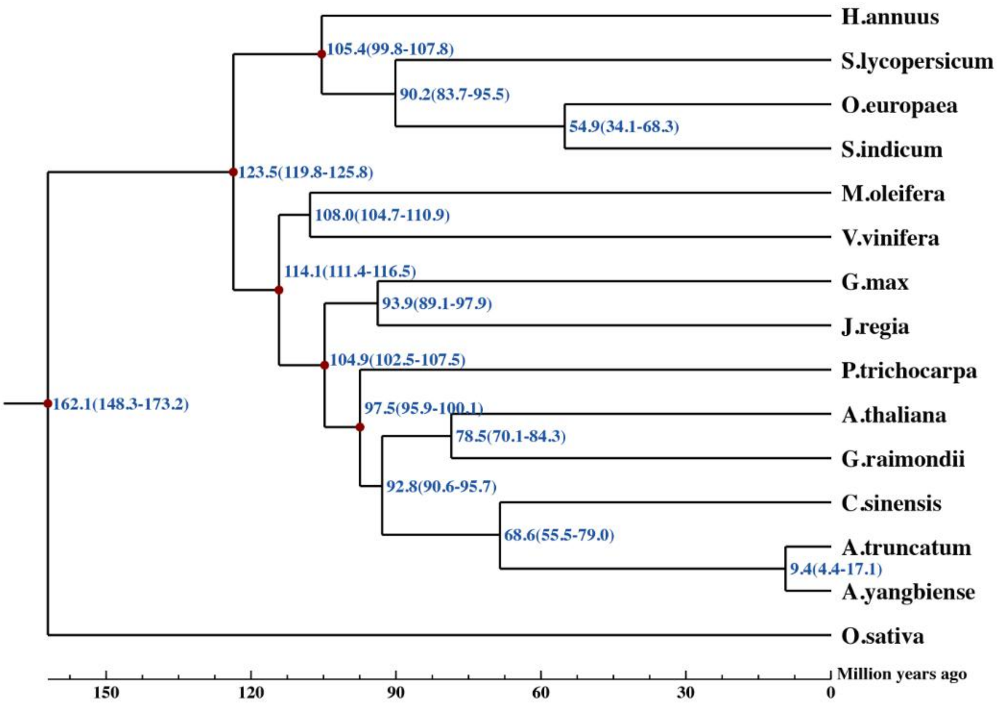

Figure S5. The phylogeny and divergence time of *A. truncatum* and 14 other plants.

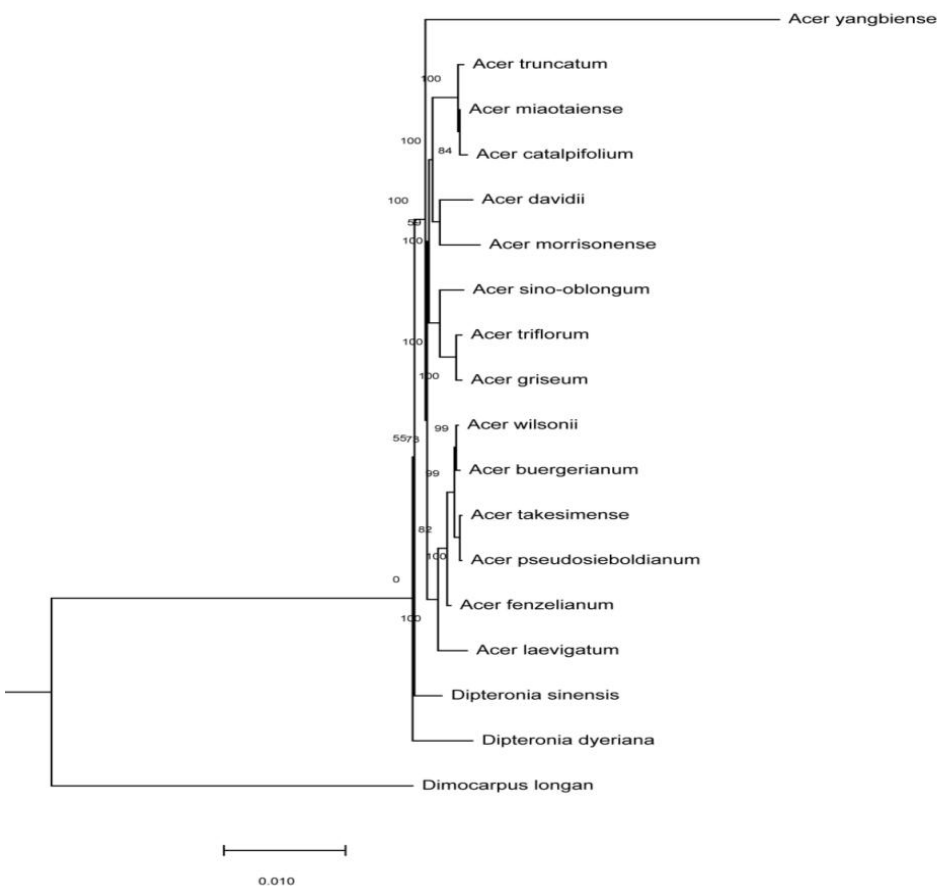

Figure S6. The phylogeny based on coding sequence of single-copy orthologous gene shared among 17 chloroplast genomes in Aceraceae species, *Dimocarpus longan* served as the outgroup.

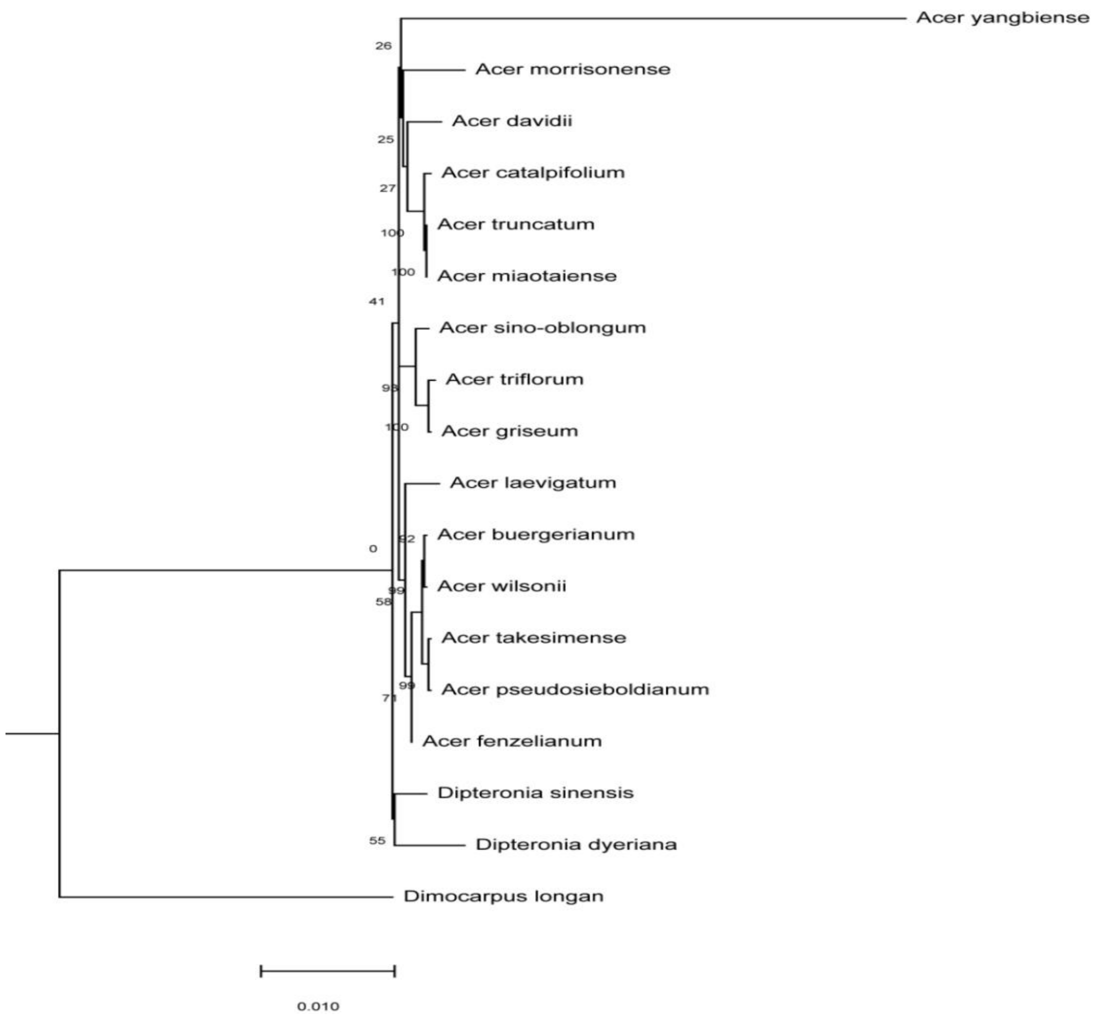

Figure S7. The phylogeny based on protein sequences of single-copy orthologous gene shared among 17 chloroplast genomes in Aceraceae species, *Dimocarpus longan* served as the outgroup.

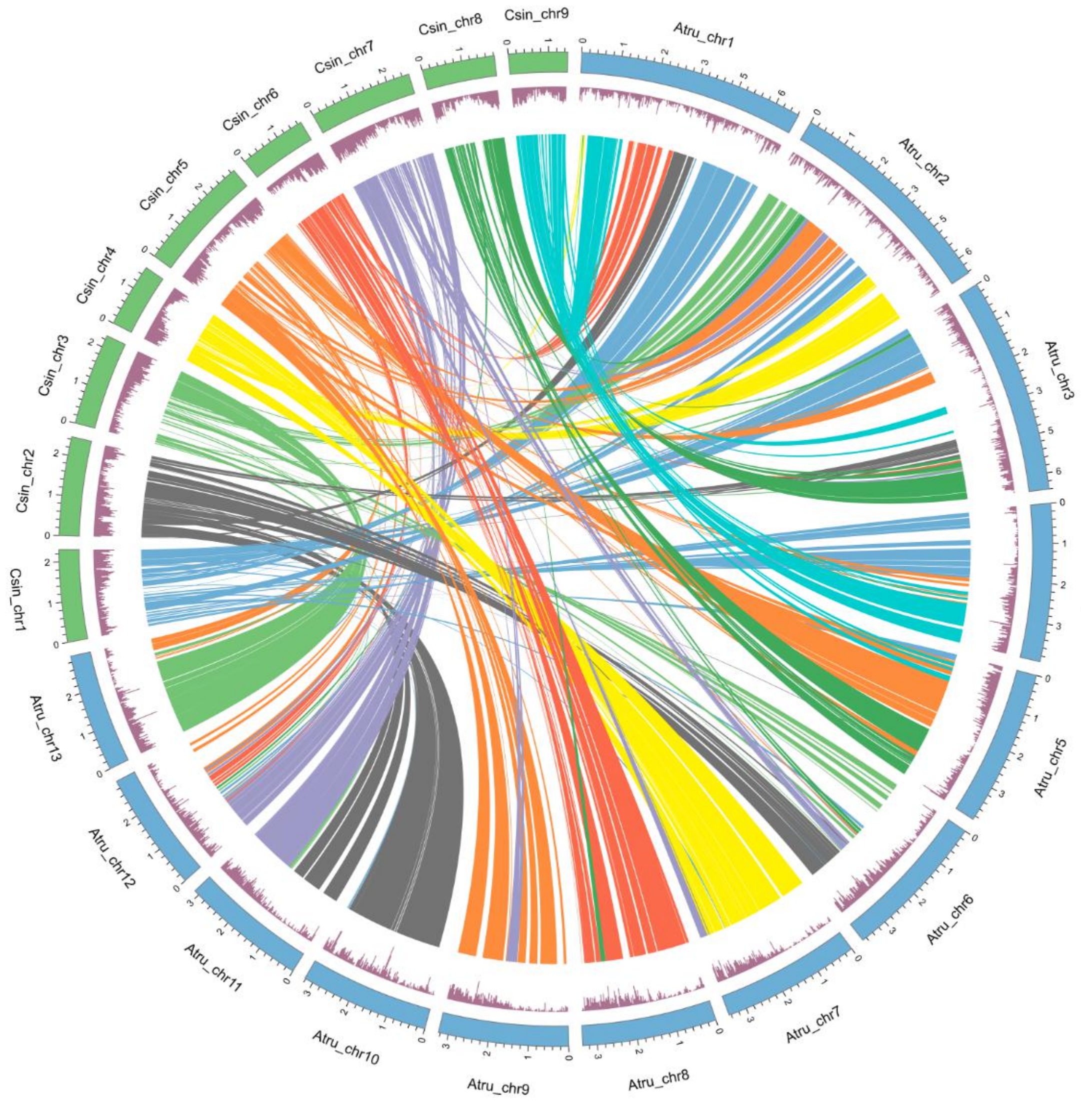

Figure S8. Synteny comparison of *A. truncatum* and *C. sinensis*. The outmost circle (blue) displays ideograms of the pseudochromosomes of the genomes. Atru: *A. truncatum*, Csin: *C. sinensis*. The second circle displays gene density.The innermost circle displays homologies between *A. truncatum* and *C. sinensis.* All distributions are drawn in a window size of 300 kb, chromosomes_units = 500000.

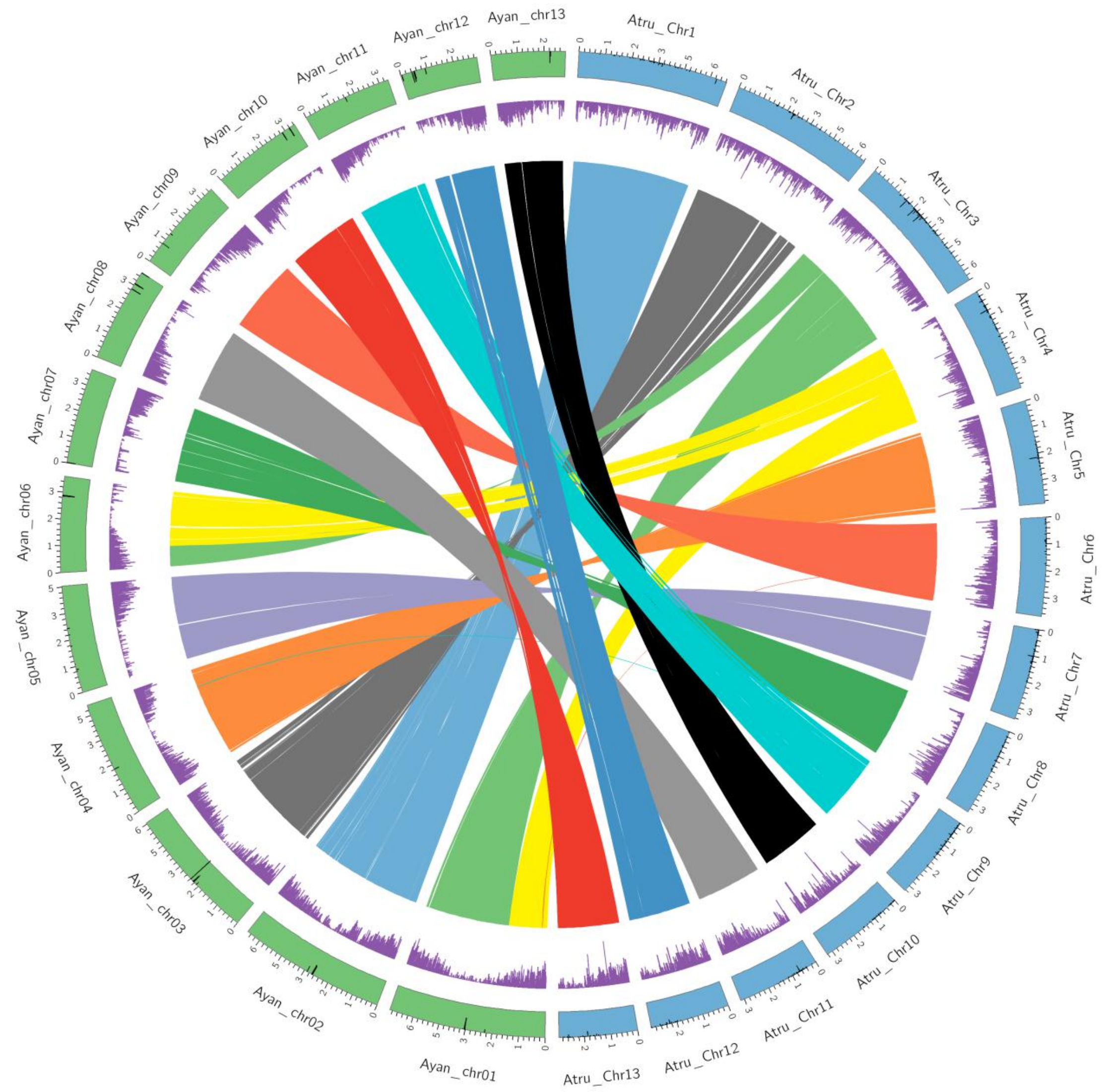

Figure S9. Synteny comparison of *A. truncatum* and *A. yangbiense*. The outmost circle (blue) displays ideograms of the pseudochromosomes of the genomes. Atru: *A. truncatum***,** Ayan: *A. yangbiense*, the narrow peaks represent putative centromeric regions. The second circle displays gene density.The innermost circle displays homologies between *A. truncatum* and *A. yangbiense.* All distributions are drawn in a window size of 300 kb, chromosomes_units = 500000.

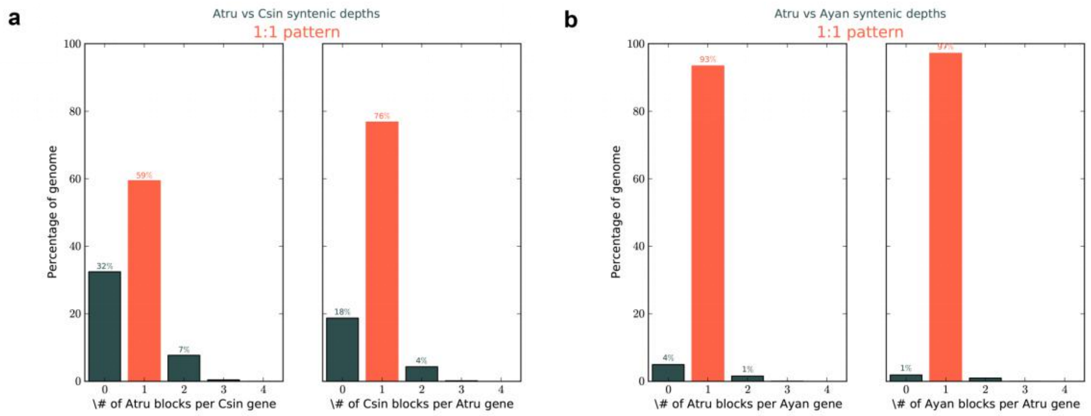

Figure S10. Syntenic pattern of *A. truncatum* with *A. yangbiense* and *C. sinensis*. The x-axis represents that each gene of one genome has no orthologous region, one orthologous region ,two orthologous regions, three orthologous regions and four orthologous regions with another genome. The y-axis is the percentage of the gene blocks in genomes. Atru: *A. truncatum* , Ayan: *A. yangbiense* ; Csin*: C. sinensis.*

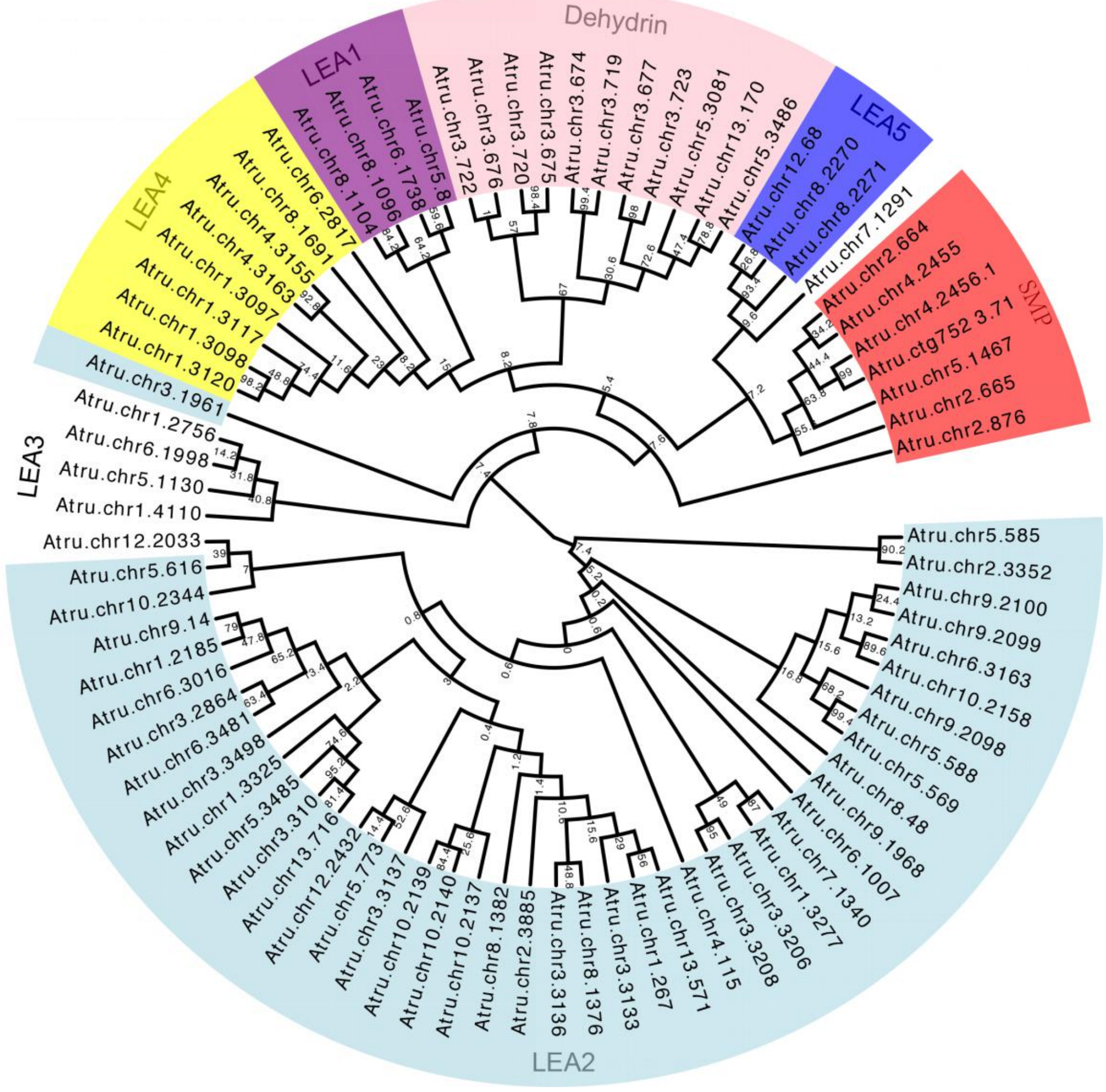

Figure S11. The phylogeny analysis of *LEA* genes in *A. truncatum*.

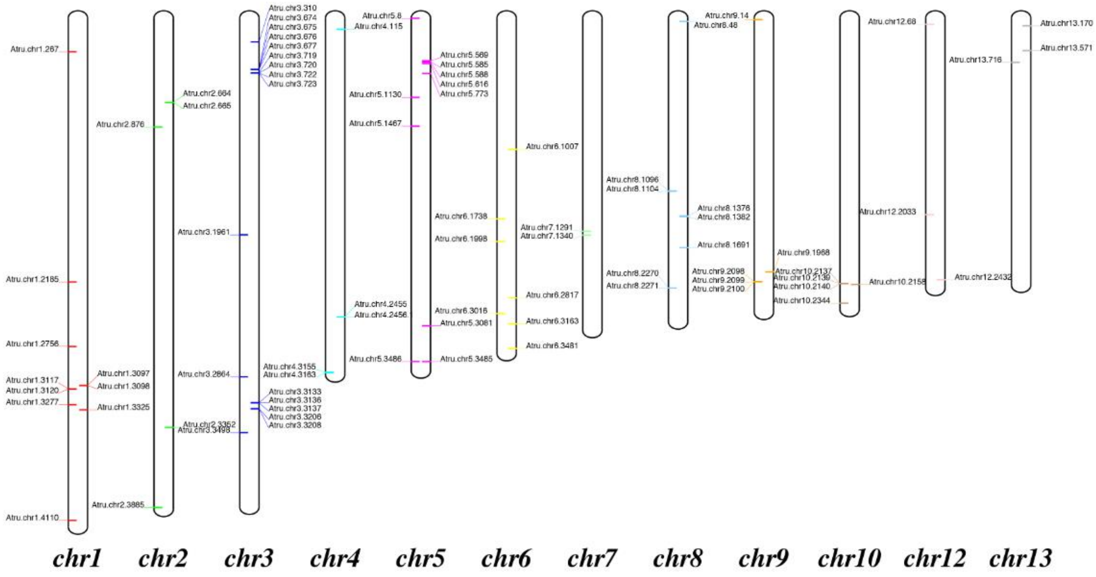

Figure S12. The distribution of *LEA* genes in *A. truncatum*.

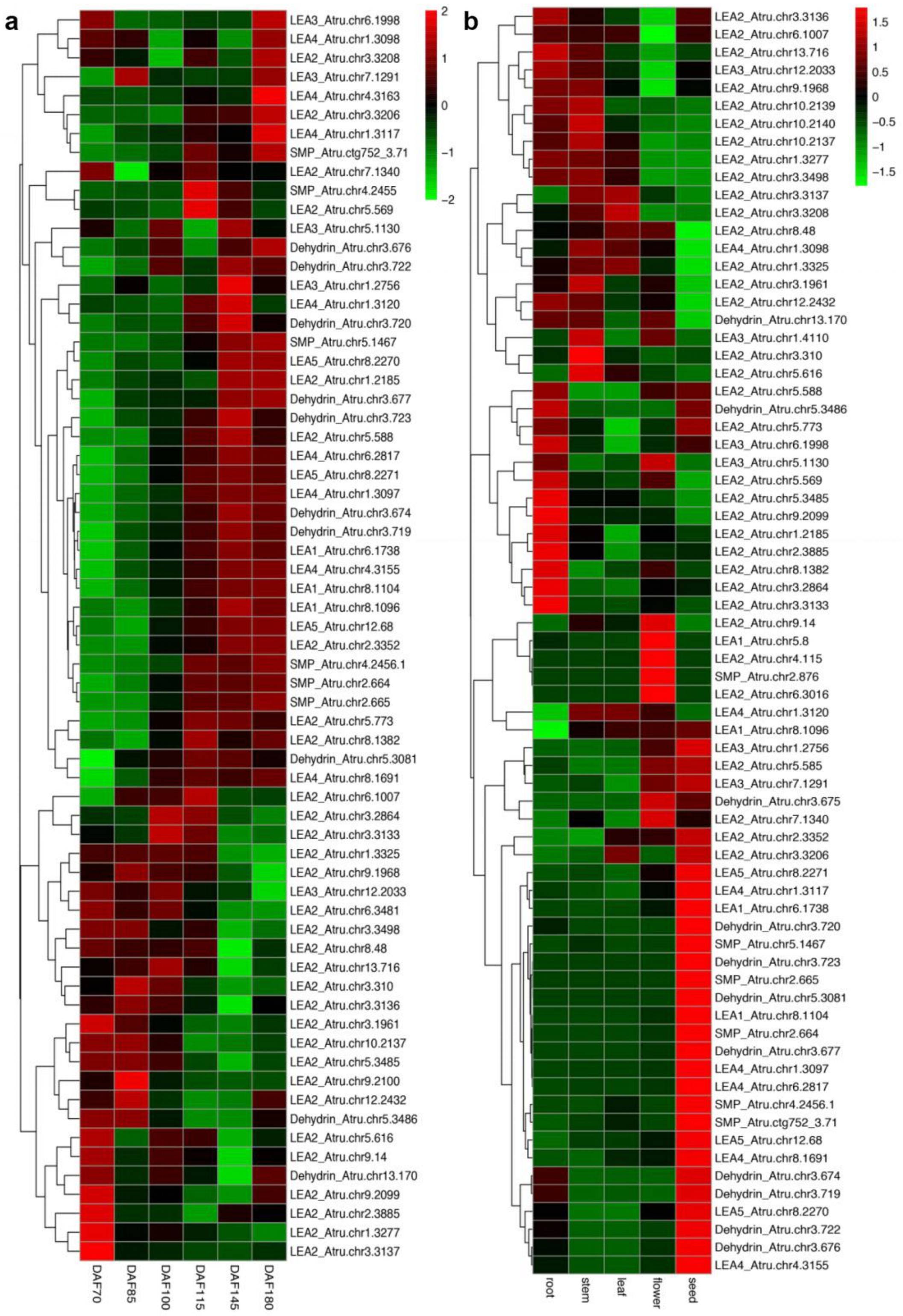

Figure S13. Heat map of gene expression profiles of *LEA* genes. Left: Differential expression *LEA* genes during seed development times; Right: Differential expression *LEA* genes in five tissues.

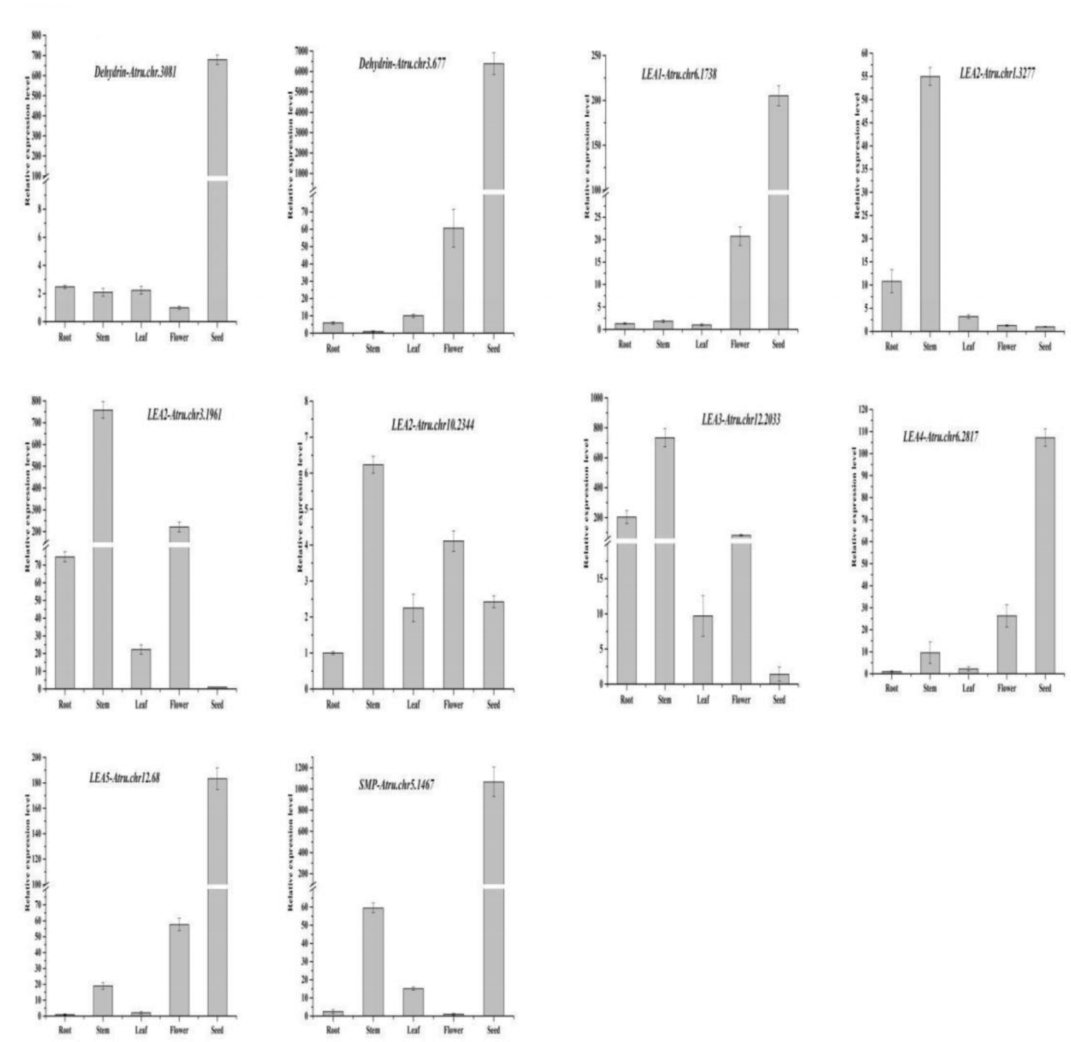

Figure S14. Quantitative PCR of *LEA* genes in different tissues. Error bars are standard errors of the mean from three technical replicates.

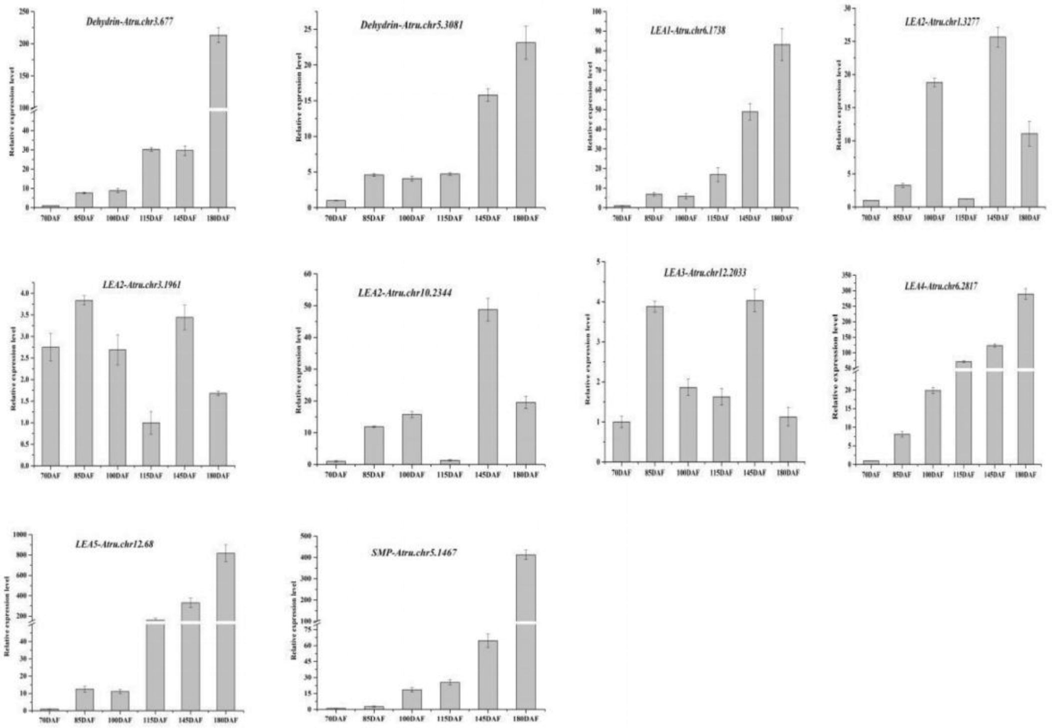

Figure S15. Quantitative PCR of *LEA* genes in six seed-development stages. Error bars are standard errors of the mean from three technical replicates.

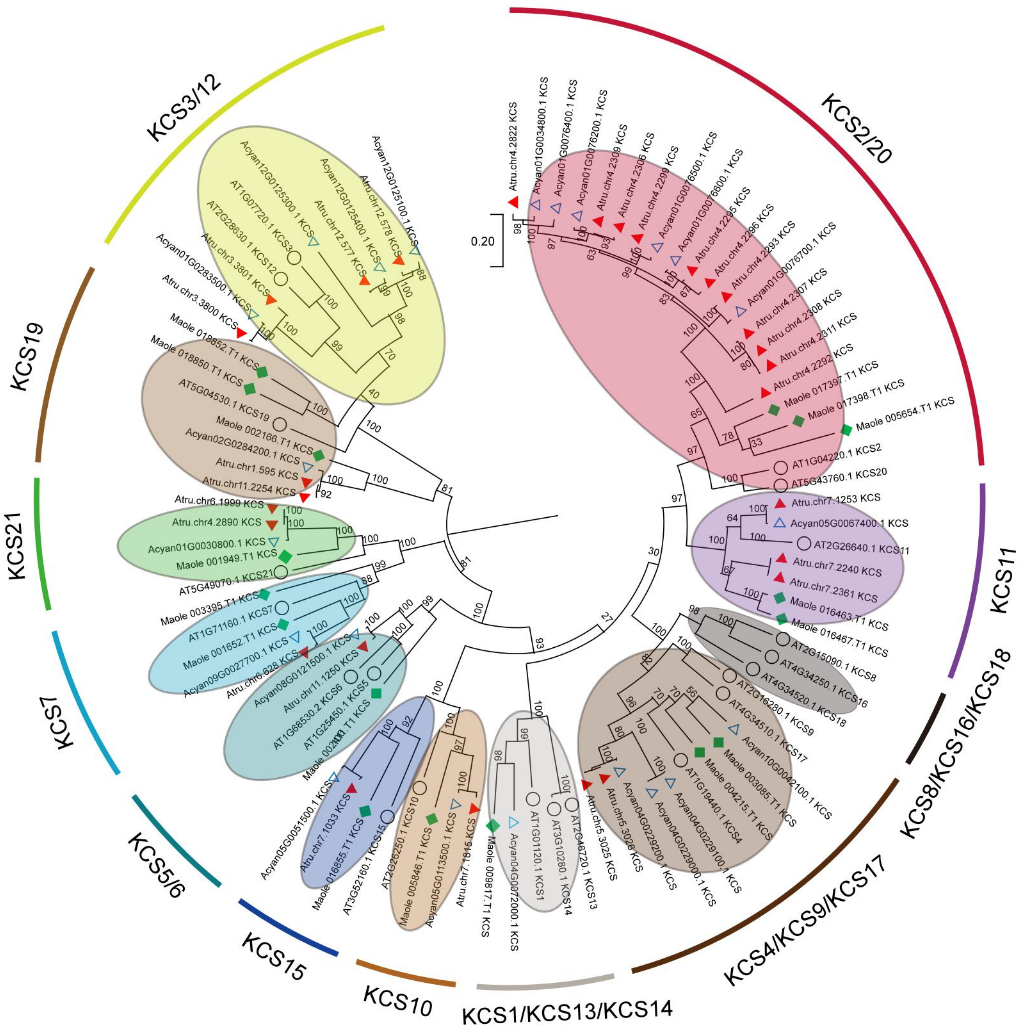

Figure S16. The phylogenetic tree and distribution of *KCS* genes based on coding sequences of *A. truncatum*. The phylogenetic tree and distribution of *KCS* genes. (a)The ML tree of *KCS* genes. *A. truncatum* (red triangle), *A. yangbiense* (blue triangle), *M. oleifera* (green square), *A. thaliana* (write circle).

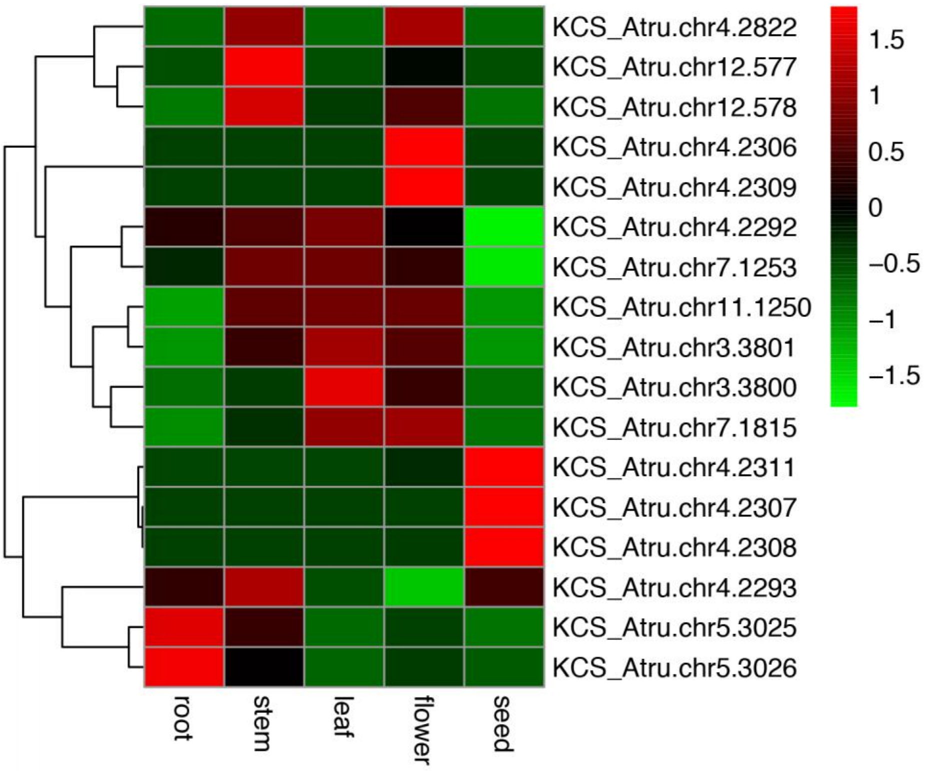

Figure S17. The expression profiles of *KCS* genes in different tissues of *A. truncatum*. Z-scores were calculated after log10 transformation of FPKM values plus 1.

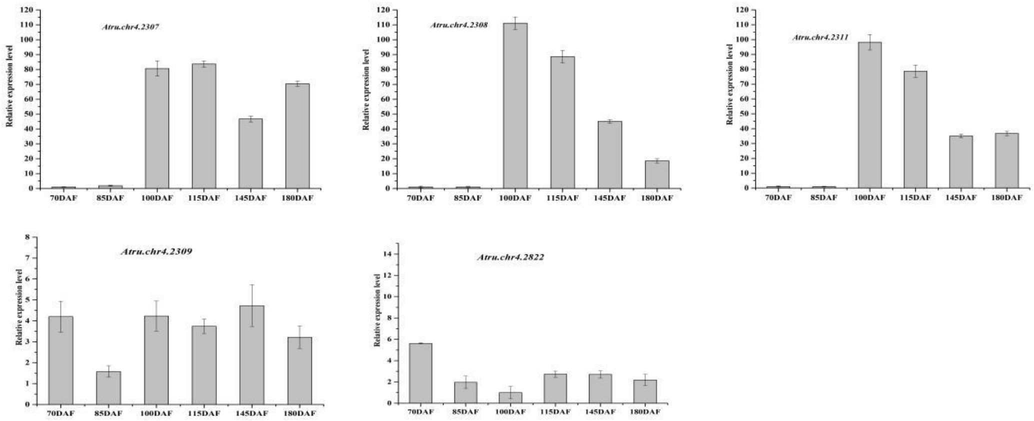

Figure S18. Quantitative PCR of *KCS* genes in six seed-development stages. Error bars are standard errors of the mean from three technical replicates.

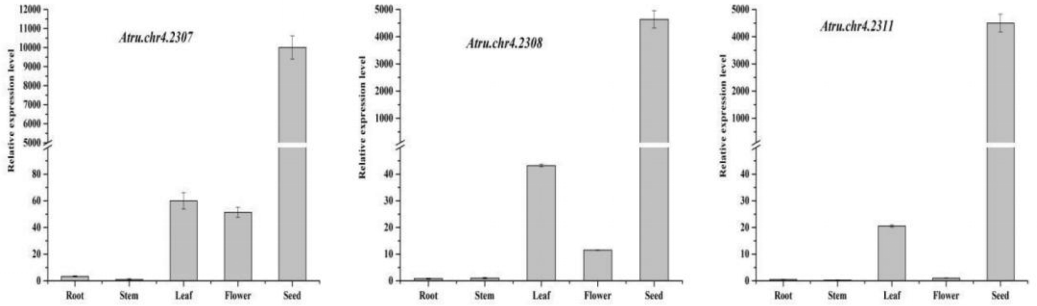

Figure S19. Quantitative PCR of *KCS* genes in different tissues. Error bars are standard errors of the mean from three technical replicates.

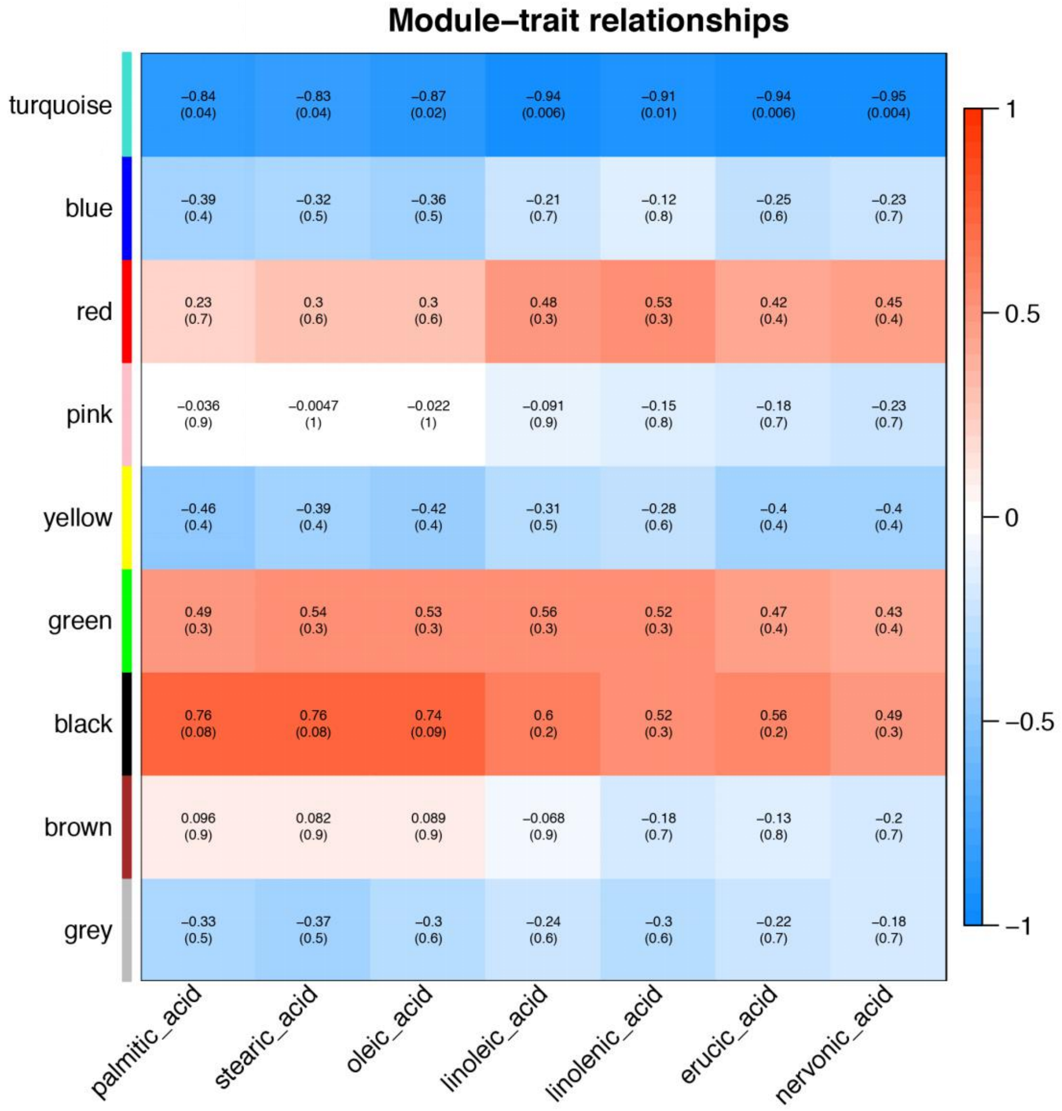

Figure S20. WGCNA analysis of fatty acid contents with RNA-seq data of seed development stages in *A. truncatum*. The numbers within the heat map represent correlations (red, positively correlated, blue, negatively correlated) and P-values (in parentheses) for the module-trait associations.
