# Supplementary material for: The Acer truncatum genome provides insights into nervonic acid biosynthesis
Source: Plant J. 2020 Oct 2;104(3):662–78. doi: 10.1111/tpj.14954 (PMC7702125; doi:10.1111/tpj.14954)
Supplement: Supplementary file 2 — Table S1. K‐mer statistics of A. truncatum. Table S2. Sequencing data of A. truncatum. Table S3. Summary of A. truncatum pseudomolecules. Table S4. The Hi‐C assembly results of A. truncatum. Table S5. Coverage statistics of the A. truncatum genome. Table S6. CEGMA evaluation results. Table S7. BUSCO notation assessment of A. truncatum genome and annotation. Table S8. Assessing the gene region assembly by mapping the RNA assembly to the A. truncatum genome. Table S9. Statistical results of gene functional annotations. Table S10. Statistical results of non‐coding RNAs. Table S11. Classification of repetitive elements in the A. truncatum genome. Table S12. The LTR subfamilies in A. truncatum and other published genomes. Table S13. The seed sequences of centromeric regions of A. truncatum and A. yangbiense chromosomes. Table S14. Details of the chloroplast genome sequences used for the phylogenetic analysis. Table S15. GO enrichment results for expanded genes in A. truncatum. Table S16. GO enrichment results for contracted genes in A. truncatum. Table S17. GO enrichment results for the genes in A. truncatum‐specific gene families. Table S18. KEGG enrichment results for expanded genes in A. truncatum. Table S19. GO enrichment results for expanded genes in A. yangbiense. Table S20. GO enrichment results for contracted genes in A. yangbiense. Table S21. KEGG enrichment results for expanded genes in A. yangbiense. Table S22. A summary of drought‐tolerant genes identified in A. truncatum and other published genomes. Table S23. A summary of drought tolerance‐related transcription factors identified in A. truncatum and other published genomes. Table S24. The copy numbers of VLCFA biosynthesis‐related genes in 12 plants. Table S25. Expression levels of VLCFA biosynthesis‐related genes that are significantly upregulated between 85 DAF and 100 DAF and are involved in fatty acid elongation. Table S26. The transcription factors (TFs)/transcription regulatory factors (TRs) in the bl [file TPJ-104-662-s002.pdf]

# The *Acer truncatum* genome provides insights into the nervonic acid biosynthesis

## Supplementary information

### This document includes the following:
### - 28 supplementary tables

**Supplementary Table Legends**

**Table S1. K-mer statistics of *A. truncatum.***

**Table S2. Sequencing data of *A. truncatum*.**

**Table S3. Summary of *A. truncatum* pseudomolecules.**

**Table S4. The Hi-c assemble results of *A. truncatum*.**

**Table S5. Coverage statistics of the *A. truncatum* genome.**

**Table S6. CEGMA evaluation results.**

**Table S7. BUSCO notation assessment of *A. truncatum* genome and annotation.**

**Table S8. Assessing the gene region assembly by mapping RNA assembly to *A. truncatum* genome.**

**Table S9. Statistical results of gene functional annotations.**

**Table S10. Statistical results of non-coding RNAs.**

**Table S11. Classification of repetitive elements in the *A. truncatum* genome.**

**Table S12. The LTR subfamilies in *A. truncatum* and other published genomes.**

**Table S13. The seed sequences of centromeric regions of *A. truncatum* and *A. yangbiense* chromosomes.**

**Table S14. Details of the chloroplast genome sequences used for the phylogenetic analysis.**

**Table S15. Go enrichment results for expanded genes in *A. truncatum.***

**Table S16. Go enrichment results for contracted genes in *A. truncatum.***

**Table S17. Go enrichment results for the genes in *A. truncatum*-spcific gene families.**

**Table S18. KEGG enrichment results for expanded genes in *A. truncatum.***

**Table S19. Go enrichment results for expanded genes in *A. yangbiense*.**

**Table S20. Go enrichment results for contracted genes in *A. yangbiense*.**

**Table S21. KEGG enrichment results for expanded genes in *A. yangbiense*.**

**Table S22. A summary of Drought-tolerant genes identified in *A. truncatum* and other published genomes.**

**Table S23. A summary of drought-tolerant transcription fators identified in *A. truncatum* and other published genomes.**

**Table S24. The gene copy number of VLCFA biosynthesis in 12 plants.**

**Table S25. Expression levels significantly up-regulated between 85 DAF and 100DAF in the fatty acid elongation stage of the VLCFA biosynthesis pathway.**

**Table S26. The transcription factors (TF)/transcription regulatory factors (TR) in black module of WGCNA analysis.**

**Table S27. List of *KCS* gene-specific primer (5' - 3') used for real-time RT-PCR.**

**Table S28. List of *LEA* gene-specific primer (5' - 3') used for real-time RT-PCR.**

**Table S1. K-mer statistics of *A. truncatum*.**

| K-mer | K-mer number | K-mer Depth | Genome Size (Mbp) | Revised Genome Size (Mbp) | Heterozygous Ratio (%) | Repeat (%) |
|---|---|---|---|---|---|---|
| 17 | 50,317,377,088 | 76 | 662.08 | 653.44 | 1.12 | 61.70 |

**Table S2. Sequencing data of *A. truncatum*.**

| Paired-end libraries | Insert size | Total data (G) | Sequence coverage (X)* |
|---|---|---|---|
| Illumina reads | 350 bp | 117.93 | 180.48 |
| PacBio long reads | 20kb | 75.7 | 115.85 |
| 10X Genomics | - | 81.54 | 124.79 |
| Hi-C reads | - | 72.21 | 110.51 |
| Total | - | 347.38 | 531.63 |

* 653.44M genome size estimated by survey.

**Table S3. Summary of *A. truncatum* pseudomolecules.**

| Chromosome | Length(bp) | Gene number | Gap base (N) |
|---|---|---|---|
| chr1 | 70,314,080 | 2,754 | 463,938 |
| chr2 | 67,888,635 | 2,427 | 647,076 |
| chr3 | 67,579,682 | 2,985 | 442,334 |
| chr4 | 49,301,835 | 2,029 | 375,130 |
| chr5 | 48,765,855 | 2,619 | 250,582 |
| chr6 | 46,359,076 | 2,521 | 369,439 |
| chr7 | 43,170,434 | 1,920 | 268,073 |
| chr8 | 41,982,645 | 1,871 | 160,723 |
| chr9 | 40,653,649 | 1,864 | 248,943 |
| chr10 | 40,269,369 | 1,570 | 423,772 |
| chr11 | 39,201,709 | 1,954 | 280,111 |
| chr12 | 37,356,478 | 1,851 | 263,834 |
| chr13 | 36,914,844 | 1,925 | 244,204 |
| Total | 629,758,291 (99.44%) | 28,290 (99.48%) | 4,448,159 |

The rest of scaffolds unanchored to chromosome are not listed.

**Table S4. The Hi-c assemble results of *A.truncatum*.**

| | length | | number | |
|---|---|---|---|---|
| | Contig**(bp) | Scaffold (bp) | Contig** | Scaffold |
| Total | 628,843,326 | 633,281,485 | 1,453 | 34 |
| Max | 4,570,384 | 70,314,080 | — | — |
| Number > = 2000 | — | — | 1,453 | 34 |
| N50 | 773,169 | 46,359,076 | 239 | 6 |
| N60 | 605,964 | 43,170,434 | 330 | 7 |
| N70 | 455,890 | 40,653,649 | 450 | 9 |
| N80 | 321,326 | 40,269,369 | 614 | 10 |
| N90 | 196,874 | 37,356,478 | 859 | 12 |

**Contig after scaffolding

**Table S5. Coverage statistics of the *A. truncatum* genome.**

| - | Percentage |
|---|---|
| Mapping rate (%) | 97.24 |
| Coverage (%) | 99.47 |
| Coverage at least 4X (%) | 99.34 |
| Coverage at least 10X (%) | 99.25 |
| Coverage at least 20X (%) | 99.11 |

Average sequence depth: The average depth of each base in the genome that is covered by the reads. Coverage: The proportion of the genome covered by the reads.

**Table S6. CEGMA evaluation results.**

| Complete | | Complete + partial | |
|---|---|---|---|
| # Prots | % Completeness | # Prots | % Completeness |
| 232 | 93.55 | 239 | 96.37 |

Complete: core genes that were completely assembled with identity greater than 0.7.

Partial: core genes that were assembled but not complete.

# Prots: the number of core genes.

% Completeness: percentage of assembled core genes to core genes.

**Table S7. BUSCO notation assessment of *A. truncatum* genome and annotation.**

| BUSCO | Genome | | Proteins | |
|---|---|---|---|---|
| | Count | Ratio(%) | Count | Ratio(%) |
| Complete BUSCOs | 1342 | 93.2 | 1395 | 96.9 |
| Complete sigle-copy BUSCOs | 1275 | 88.5 | 1240 | 86.1 |
| Complete duplicated BUSCOs | 68 | 4.7 | 155 | 10.8 |
| Fragmented BUSCOs | 18 | 1.3 | 30 | 2.1 |
| Missing BUSCOs | 79 | 5.5 | 15 | 1 |
| Total BUSCO group serarched | 1440 | | 1440 | |

**Table S8. Assessing the gene region assembly by mapping RNA assembly to *A. truncatum* genome.**

| Dataset | Number | Total length (bp) | Sequences covered by assembly (%) | with >90% sequence in one scaffold | | with >50% sequence in one scaffold | |
|---|---|---|---|---|---|---|---|
| | | | | Number | Percent | Number | Percent |
| >500bp | 106,055 | 178,432,686 | 95.97 | 94,327 | 88.94 | 100,924 | 95.16 |
| >1000bp | 67,579 | 151,355,839 | 99.40 | 62,942 | 93.14 | 66,919 | 99.02 |
| >2000bp | 31,334 | 98,869,172 | 99.77 | 29,229 | 93.28 | 31,133 | 99.36 |
| >5000bp | 2,133 | 13,361,783 | 99.86 | 1,911 | 89.59 | 2,107 | 98.78 |

**Table S9. Statistical results of gene functional annotations.**

| | Number | Percent (%) |
|---|---|---|
| Total | 28,438 | - |
| Swiss-Prot | 21,289 | 74.86 |
| NR | 27,202 | 95.65 |
| KEGG | 21,276 | 74.81 |
| IterPro | 27,826 | 97.84 |
| GO | 25,432 | 89.43 |
| Pfam | 21,579 | 75.87 |
| Annotated | 28,023 | 98.54 |
| Un-annotated | 415 | 0.01 |

**Table S10. Statistical results of non-coding RNAs.**

| Type | | Copy | Average length (bp) | Total length (bp) | (% ) of genome |
|---|---|---|---|---|---|
| miRNA | | 1,345 | 132.48 | 178,186 | 0.028137 |
| tRNA | | 744 | 75.11 | 55,883 | 0.008824 |
| rRNA | rRNA | 368 | 169.31 | 62,307 | 0.009839 |
| | 18S | 54 | 497.61 | 26,871 | 0.004243 |
| | 28S | 42 | 125.14 | 5,256 | 0.00083 |
| | 5.8S | 15 | 123.46 | 1,852 | 0.000292 |
| | 5S | 257 | 110.23 | 28,328 | 0.004473 |
| snRNA | snRNA | 868 | 113 | 98,086 | 0.015489 |
| | CD-box | 655 | 102.54 | 67,164 | 0.010606 |
| | HACA-box | 51 | 139/82 | 7,131 | 0.001126 |
| | Splicing | 160 | 147.04 | 23,526 | 0.003715 |

**Table S11. Classification of repetitive elements in the *A. truncatum* genome.**

| Type | Length (bp) | Percentage of genome (%) |
|---|---|---|
| DNA | 48,529,125 | 7.66 |
| LINE | 33,244,458 | 5.24 |
| SINE | 281,678 | 0.04 |
| LTR | 287,494,231 | 45.4 |
| LTR/Copia | 163,812,683 | 25.87 |
| LTR/Gypsy | 99,823,924 | 15.76 |
| Tandem repeats | 29,043,401 | 4.59 |
| Satellite | 1,088,584 | 0.17 |
| Simple repeats | 4,660,170 | 0.74 |
| Unknown | 20,698,204 | 3.27 |
| Total | 391,045,082 | 61.75 |

The total length of all repetitive elements is the result of removing redundancy

**Table S12. The LTR subfamilies in *A. truncatum* and other published genomes.**

| superfamily | family | number in *A. truncatum* | number in *A.yangbiense* | number in *C. sinensis* | number in *M. oleifera* |
|---|---|---|---|---|---|
| Ty3/Gypsy | reina | 260 | 210 | 161 | 57 |
| Ty3/Gypsy | tat | 1,789 | 1,831 | 271 | 1,162 |
| Ty3/Gypsy | athila | 1,094 | 676 | 751 | 2,045 |
| Ty3/Gypsy | crm | 506 | 463 | 118 | 544 |
| Ty3/Gypsy | galadriel | 36 | 23 | 48 | 928 |
| Ty3/Gypsy | del | 13 | 10 | 62 | 10,005 |
| Ty1/Copia | sire | 1,727 | 6,624 | 321 | 8,373 |
| Ty1/Copia | tork | 2,200 | 1,772 | 668 | 2,379 |
| Ty1/Copia | retrofit | 1,269 | 924 | 425 | 1,838 |
| Ty1/Copia | oryco | 262 | 212 | 103 | 498 |
| Caulimoviridae | caulimovirus | 43 | 96 | 181 | 94 |
| Caulimoviridae | badnavirus | 8 | 11 | 27 | 8 |
| Caulimoviridae | soymovirus | 2 | 1 | 6 | 1 |
| Caulimoviridae | cavemovirus | 0 | 2 | 5 | 8 |
| Unclassified | | 835 | 661 | 47 | 125 |
| **Total** | | **10,044** | **13,516** | **3,194** | **28,065** |

**Table S13. The seed sequences of putative centromeric regions of *A. truncatum* and *A. yangbiense* chromosomes.**

| Species | Monomer sequence | Length | GC(%) |
|---|---|---|---|
| *A. truncatum* | GAAGTAAGGAACCAGGTACCCCGATGAGGCATCCCGATCTTA<br>CTGTAAGTCTGTCCTGGTAGGCAACTAAGGCCACTTTAGCACC<br>AGGCTTCTTGGAGGAGGGTATTCCCCGACCAAGGCAGCAGGA<br>GCGCCATAAGGCTATTTCCTGGTCTTGAAATGGC | 161 | 53 |
| *A. yangbiense* | CTTTTTGGAATGCAAACTTTCTTTTTGGCATGCGGGCATGCTTT<br>TTTGAATGCGAGAGTGGTTTTTAGCTCTCGAGCGTGCTTTTTA<br>GTTTCCAAACGTTCTTTTTGGCTTTCGGGCGTTCTTTTTTGCAA<br>GCAAACTTTCTTTTTGGCTTTTGGGCGTT | 160 | 41.25 |

**Table S14. Details of the chloroplast genome sequences used for the phylogenetic analysis.**

| No. | Taxon | Family | Order | GenBank Accession number |
|---|---|---|---|---|
| 1 | *Acer yangbiense* | Aceraceae | Sapindales | MK479229 |
| 2 | *Acer wilsonii* | Aceraceae | Sapindales | NC_040988 |
| 3 | *Acer triflorum* | Aceraceae | Sapindales | NC_047296 |
| 4 | *Acer takesimense* | Aceraceae | Sapindales | NC_046488 |
| 5 | *Acer sino-oblongum* | Aceraceae | Sapindales | NC_040106 |
| 6 | *Acer pseudosieboldianum* | Aceraceae | Sapindales | NC_046487 |
| 7 | *Acer laevigatum* | Aceraceae | Sapindales | NC_042443 |
| 8 | *Acer fenzelianum* | Aceraceae | Sapindales | NC_045527 |
| 9 | *Acer catalpifolium* | Aceraceae | Sapindales | MK479218 |
| 10 | *Dipteronia dyeriana* | Sapindaceae | Sapindales | NC_031899 |
| 11 | *Dipteronia sinensis* | Sapindaceae | Sapindales | NC_029338 |
| 12 | *Acer buergerianum* | Aceraceae | Sapindales | NC_034744 |
| 13 | *Acer davidii* | Aceraceae | Sapindales | NC_030331 |
| 14 | *Acer griseum* | Aceraceae | Sapindales | NC_034346 |
| 15 | *Acer miaotaiense* | Aceraceae | Sapindales | NC_030343 |
| 16 | *Acer morrisonense* | Aceraceae | Sapindales | KT970611 |
| 17 | *Acer truncatum* | Aceraceae | Sapindales | MH638284 |
| 18 | *Dimocarpus longan* | Sapindaceae | Sapindales | NC_037447 |

**Table S15. Go enrichment results for expanded genes in *A. truncatum.***

| GO_ID | GO_Term | GO_Class | Pvalue | AdjustedPv | Gene_Number |
|---|---|---|---|---|---|
| GO:0005515 | protein binding | MF | 0.001959647 | 0.040608303 | 299 |
| GO:0016705 | oxidoreductase activity, acting on paired donors, with incorporation or reduction of molecular oxygen | MF | 0.001401486 | 0.032359948 | 61 |
| GO:0043531 | ADP binding | MF | 3.38831E-66 | 6.10235E-63 | 56 |
| GO:0016746 | transferase activity, transferring acyl groups | MF | 9.09257E-11 | 4.09393E-08 | 48 |
| GO:0030246 | carbohydrate binding | MF | 6.41451E-37 | 5.77627E-34 | 46 |
| GO:0016747 | transferase activity, transferring acyl groups other than amino-acyl groups | MF | 1.80621E-14 | 1.08433E-11 | 42 |
| GO:0044283 | small molecule biosynthetic process | BP | 0.000187938 | 0.007051585 | 37 |
| GO:0046394 | carboxylic acid biosynthetic process | BP | 3.0831E-05 | 0.001914714 | 36 |
| GO:0046983 | protein dimerization activity | MF | 0.001604763 | 0.03628026 | 31 |
| GO:0005618 | cell wall | CC | 2.47237E-07 | 4.94748E-05 | 22 |
| GO:0006631 | fatty acid metabolic process | BP | 7.6302E-06 | 0.000654381 | 22 |
| GO:0006633 | fatty acid biosynthetic process | BP | 9.51125E-07 | 0.000171298 | 21 |
| GO:0005976 | polysaccharide metabolic process | BP | 0.001631705 | 0.03628026 | 21 |
| GO:0044264 | cellular polysaccharide metabolic process | BP | 0.000204111 | 0.00750212 | 20 |
| GO:0006073 | cellular glucan metabolic process | BP | 4.57111E-05 | 0.002007944 | 19 |
| GO:0004983 | neuropeptide Y receptor activity | MF | 0.00161711 | 0.03628026 | 14 |
| GO:0016903 | oxidoreductase activity, acting on the aldehyde or oxo group of donors | MF | 3.44434E-06 | 0.000345239 | 13 |
| GO:0000160 | phosphorelay signal transduction system | BP | 3.84716E-05 | 0.002007944 | 13 |
| GO:0044703 | multi-organism reproductive process | BP | 0.000406762 | 0.011099665 | 12 |
| GO:0016762 | xyloglucan:xyloglucosyl transferase activity | MF | 9.98602E-09 | 2.2481E-06 | 11 |
| GO:0048046 | apoplast | CC | 9.98602E-09 | 2.2481E-06 | 11 |
| GO:0004176 | ATP-dependent peptidase activity | MF | 3.40201E-09 | 1.02117E-06 | 10 |
| GO:0008408 | 3'-5' exonuclease activity | MF | 8.64985E-06 | 0.000688866 | 9 |
| GO:0010333 | terpene synthase activity | MF | 0.000173781 | 0.006659156 | 9 |
| GO:0009067 | aspartate family amino acid biosynthetic process | BP | 0.000173781 | 0.006659156 | 9 |
| GO:0016570 | histone modification | BP | 0.000214961 | 0.007591075 | 9 |
| GO:0048544 | recognition of pollen | BP | 0.000263928 | 0.00833919 | 9 |
| GO:0043624 | cellular protein complex disassembly | BP | 0.001961645 | 0.040608303 | 9 |
| GO:0080019 | fatty-acyl-CoA reductase (alcohol-forming) activity | MF | 7.13271E-10 | 2.5692E-07 | 8 |
| GO:0030042 | actin filament depolymerization | BP | 2.21586E-06 | 0.000249422 | 8 |

| | | | | | |
|---|---|---|---|---|---|
| GO:0003871 | 5-methyltetrahydropteroyltriglutamate-homocysteine S-methyltransferase activity | MF | 3.64216E-06 | 0.000345239 | 8 |
| GO:0009086 | methionine biosynthetic process | BP | 5.77669E-06 | 0.000520191 | 8 |
| GO:0009678 | hydrogen-translocating pyrophosphatase activity | MF | 1.94249E-06 | 0.000249422 | 7 |
| GO:0004523 | RNA-DNA hybrid ribonuclease activity | MF | 1.94249E-06 | 0.000249422 | 7 |
| GO:0003950 | NAD+ ADP-ribosyltransferase activity | MF | 1.11834E-05 | 0.000805655 | 7 |
| GO:0004427 | inorganic diphosphatase activity | MF | 4.25544E-05 | 0.002007944 | 7 |
| GO:0030321 | transepithelial chloride transport | BP | 0.002199364 | 0.043053925 | 7 |
| GO:0008113 | peptide-methionine (S)-S-oxide reductase activity | MF | 2.01358E-06 | 0.000249422 | 6 |
| GO:0031625 | ubiquitin protein ligase binding | MF | 3.39559E-05 | 0.001972728 | 6 |
| GO:0031461 | cullin-RING ubiquitin ligase complex | CC | 0.000403295 | 0.011099665 | 6 |
| GO:0016624 | oxidoreductase activity, acting on the aldehyde or oxo group of donors, disulfide as acceptor | MF | 0.000288205 | 0.008509136 | 5 |
| GO:0005730 | nucleolus | CC | 0.001787014 | 0.03924893 | 5 |
| GO:0003852 | 2-isopropylmalate synthase activity | MF | 9.17978E-06 | 0.000688866 | 4 |
| GO:0009098 | leucine biosynthetic process | BP | 4.38813E-05 | 0.002007944 | 4 |
| GO:0031429 | box H/ACA snoRNP complex | CC | 4.38813E-05 | 0.002007944 | 4 |
| GO:0006850 | mitochondrial pyruvate transport | BP | 0.000280832 | 0.008429636 | 4 |
| GO:0004602 | glutathione peroxidase activity | MF | 0.000924636 | 0.023128731 | 4 |
| GO:0000148 | 1,3-beta-D-glucan synthase complex | CC | 0.002215603 | 0.043053925 | 4 |
| GO:0006075 | (1->3)-beta-D-glucan biosynthetic process | BP | 0.002215603 | 0.043053925 | 4 |
| GO:0008241 | peptidyl-dipeptidase activity | MF | 0.000166951 | 0.006659156 | 3 |

**Table S16. Go enrichment results for contracted genes in *A. truncatum.***

| GO_ID | GO_Term | GO_Class | Pvalue | Adjusted Pvalue | Gene_Number |
|---|---|---|---|---|---|
| GO:0005488 | binding | MF | 0.001224735 | 0.015433824 | 459 |
| GO:0003824 | catalytic activity | MF | 0.000767117 | 0.012004113 | 375 |
| GO:0043167 | ion binding | MF | 6.05711E-05 | 0.001627419 | 268 |
| GO:0046872 | metal ion binding | MF | 0.000187665 | 0.004049007 | 141 |
| GO:0055114 | oxidation-reduction process | BP | 1.52291E-10 | 2.16863E-08 | 129 |
| GO:0016491 | oxidoreductase activity | MF | 4.52941E-08 | 3.22494E-06 | 128 |
| GO:0046914 | transition metal ion binding | MF | 2.36744E-05 | 0.000749162 | 96 |
| GO:0016705 | oxidoreductase activity, acting on paired donors, with incorporation or reduction of molecular oxygen | MF | 3.54928E-19 | 2.52709E-16 | 66 |
| GO:0005506 | iron ion binding | MF | 5.1146E-14 | 1.45664E-11 | 59 |
| GO:0020037 | heme binding | MF | 6.53946E-11 | 1.33031E-08 | 57 |
| GO:0009055 | electron carrier activity | MF | 7.87919E-13 | 1.86999E-10 | 50 |
| GO:0043531 | ADP binding | MF | 3.04669E-25 | 4.33848E-22 | 39 |
| GO:0004497 | monooxygenase activity | MF | 2.34938E-05 | 0.000749162 | 29 |
| GO:0016829 | lyase activity | MF | 9.46905E-11 | 1.49821E-08 | 28 |
| GO:0016757 | transferase activity, transferring glycosyl groups | MF | 0.003123367 | 0.035021064 | 28 |
| GO:0016758 | transferase activity, transferring hexosyl groups | MF | 9.03521E-05 | 0.002297524 | 26 |
| GO:0016835 | carbon-oxygen lyase activity | MF | 1.66335E-09 | 2.15328E-07 | 21 |
| GO:0010333 | terpene synthase activity | MF | 1.50937E-17 | 7.1645E-15 | 18 |
| GO:0000287 | magnesium ion binding | MF | 6.72147E-06 | 0.000258686 | 17 |
| GO:0005507 | copper ion binding | MF | 2.72163E-05 | 0.000824596 | 16 |
| GO:0006457 | protein folding | BP | 0.000136332 | 0.003081532 | 16 |
| GO:0003755 | peptidyl-prolyl cis-trans isomerase activity | MF | 1.87739E-08 | 1.59168E-06 | 15 |
| GO:0016712 | oxidoreductase activity, acting on paired donors | MF | 4.067E-05 | 0.001206545 | 13 |
| GO:0007156 | homophilic cell adhesion | BP | 0.001142326 | 0.014523858 | 13 |
| GO:0051213 | dioxygenase activity | MF | 0.004505467 | 0.048975453 | 12 |
| GO:0004650 | polygalacturonase activity | MF | 1.4332E-06 | 6.37775E-05 | 11 |
| GO:0016706 | oxidoreductase activity, acting on paired donors, with incorporation or reduction of molecular oxygen | MF | 0.001304524 | 0.016186741 | 11 |
| GO:0022414 | reproductive process | BP | 0.001458979 | 0.017606667 | 10 |
| GO:0044703 | multi-organism reproductive process | BP | 0.001609227 | 0.019096158 | 8 |

| | | | | | |
|---|---|---|---|---|---|
| GO:0008146 | sulfotransferase activity | MF | 3.38632E-09 | 4.01843E-07 | 7 |
| GO:0048544 | recognition of pollen | BP | 0.000230028 | 0.004549442 | 7 |
| GO:0008171 | O-methyltransferase activity | MF | 0.000724779 | 0.011467612 | 6 |
| GO:0010181 | FMN binding | MF | 0.003544788 | 0.038829068 | 5 |
| GO:0004144 | diacylglycerol O-acyltransferase activity | MF | 0.000109881 | 0.002565081 | 4 |
| GO:0019064 | fusion of virus membrane with host plasma membrane | BP | 0.001022891 | 0.013241788 | 4 |
| GO:0046789 | host cell surface receptor binding | MF | 0.001022891 | 0.013241788 | 4 |
| GO:0003968 | RNA-directed RNA polymerase activity | MF | 0.00132995 | 0.016186741 | 4 |
| GO:0004970 | ionotropic glutamate receptor activity | MF | 0.00132995 | 0.016186741 | 4 |
| GO:0019031 | viral envelope | CC | 0.002126459 | 0.024820311 | 4 |
| GO:0005234 | extracellular-glutamate-gated ion channel activity | MF | 0.003202222 | 0.035624716 | 4 |
| GO:0004351 | glutamate decarboxylase activity | MF | 0.000997672 | 0.013241788 | 2 |

**Table S17. GO enrichment results for the genes in *A. truncatum*-spcific gene families.**

| GO_ID | GO_Term | GO_Class | Pvalue | AdjustedPv | Gene number |
|---|---|---|---|---|---|
| GO:0008194 | UDP-glycosyltransferase activity | MF | 0.000307838 | 0.034714635 | 9 |
| GO:0035251 | UDP-glucosyltransferase activity | MF | 4.19407E-05 | 0.006148512 | 8 |
| GO:0003843 | 1,3-beta-D-glucan synthase activity | MF | 6.14017E-09 | 3.469E-06 | 7 |
| GO:0051274 | beta-glucan biosynthetic process | BP | 7.27861E-05 | 0.009700403 | 7 |
| GO:0000148 | 1,3-beta-D-glucan synthase complex | CC | 9.46522E-09 | 3.469E-06 | 6 |
| GO:0006075 | (1->3)-beta-D-glucan biosynthetic process | BP | 9.46522E-09 | 3.469E-06 | 6 |
| GO:0005730 | nucleolus | CC | 2.32706E-07 | 6.82294E-05 | 6 |
| GO:0004650 | polygalacturonase activity | MF | 0.000386561 | 0.037779935 | 6 |
| GO:0031429 | box H/ACA snoRNP complex | CC | 3.91238E-07 | 8.19363E-05 | 4 |

**Table S18. KEGG enrichment results for expanded genes in *A.truncatum*.**

| MapID | MapTitle | Pvalue | AdjustedPv | Gene_Number |
|---|---|---|---|---|
| map04626 | Plant-pathogen interaction | 7.01167E-05 | 0.000893988 | 58 |
| map04014 | Ras signaling pathway | 1.29733E-27 | 2.64655E-25 | 56 |
| map00940 | Phenylpropanoid biosynthesis | 5.46096E-10 | 2.22807E-08 | 43 |
| map00941 | Flavonoid biosynthesis | 1.20338E-25 | 1.22744E-23 | 37 |
| map00062 | Fatty acid elongation | 5.72091E-14 | 3.89022E-12 | 23 |
| map00910 | Nitrogen metabolism | 3.72291E-10 | 1.89868E-08 | 21 |
| map00073 | Cutin, suberine and wax biosynthesis | 9.48272E-08 | 1.75861E-06 | 19 |
| map00945 | Stilbenoid, diarylheptanoid and gingerol biosynthesis | 2.91522E-08 | 6.60783E-07 | 18 |
| map00460 | Cyanoamino acid metabolism | 0.00321129 | 0.019267738 | 17 |
| map00750 | Vitamin B6 metabolism | 3.52365E-09 | 1.02689E-07 | 16 |
| map00350 | Tyrosine metabolism | 0.009863451 | 0.049076685 | 13 |
| map00909 | Sesquiterpenoid and triterpenoid biosynthesis | 0.005271027 | 0.028297091 | 9 |
| map00591 | Linoleic acid metabolism | 0.000720599 | 0.005880088 | 8 |
| map00450 | Selenocompound metabolism | 0.001991129 | 0.012308799 | 8 |
| map00643 | Styrene degradation | 0.000354078 | 0.003283269 | 5 |

**Table S19. Go enrichment results for expanded genes in *A. yangbiense*.**

| GO_ID | GO_Term | GO_Class | Pvalue | AdjustedPv | Gene_Number |
|---|---|---|---|---|---|
| GO:0046914 | transition metal ion binding | MF | 2.37E-71 | 6.58E-68 | 932 |
| GO:0004803 | transposase activity | MF | 2.16E-62 | 2E-59 | 126 |
| GO:0006313 | transposition, DNA-mediated | BP | 1.23E-60 | 6.82E-58 | 131 |
| GO:0008270 | zinc ion binding | MF | 5.84E-58 | 2.7E-55 | 683 |
| GO:0043531 | ADP binding | MF | 1.4E-54 | 5.53E-52 | 201 |
| GO:0015074 | DNA integration | BP | 7.7E-53 | 2.67E-50 | 111 |
| GO:0048544 | recognition of pollen | BP | 1.19E-50 | 2.74E-48 | 122 |
| GO:0044703 | multi-organism reproductive process | BP | 3.22E-40 | 4.7E-38 | 138 |
| GO:0046872 | metal ion binding | MF | 2.99E-38 | 4.15E-36 | 1087 |
| GO:0043169 | cation binding | MF | 4.27E-37 | 5.64E-35 | 1088 |
| GO:0022414 | reproductive process | BP | 1.48E-36 | 1.87E-34 | 139 |
| GO:0005488 | binding | MF | 3.28E-33 | 3.5E-31 | 3392 |
| GO:0006310 | DNA recombination | BP | 1.05E-28 | 9.42E-27 | 176 |
| GO:0046906 | tetrapyrrole binding | MF | 1.5E-26 | 1.19E-24 | 252 |
| GO:0020037 | heme binding | MF | 9.92E-24 | 6.72E-22 | 241 |
| GO:0003676 | nucleic acid binding | MF | 5.97E-22 | 3.95E-20 | 1182 |
| GO:0016705 | oxidoreductase activity, acting on paired donors | MF | 4.89E-20 | 2.77E-18 | 224 |
| GO:0032501 | multicellular organismal process | BP | 2.31E-19 | 1.21E-17 | 196 |
| GO:0045735 | nutrient reservoir activity | MF | 4.14E-19 | 2.13E-17 | 63 |
| GO:0044707 | single-multicellular organism process | BP | 4.23E-19 | 2.13E-17 | 180 |
| GO:0006259 | DNA metabolic process | BP | 6.64E-19 | 3.29E-17 | 412 |
| GO:0097159 | organic cyclic compound binding | MF | 5.39E-18 | 2.45E-16 | 1935 |
| GO:1901363 | heterocyclic compound binding | MF | 1.32E-17 | 5.63E-16 | 1931 |
| GO:0005506 | iron ion binding | MF | 3.96E-16 | 1.64E-14 | 203 |
| GO:0016758 | transferase activity, transferring hexosyl groups | MF | 8.39E-16 | 3.38E-14 | 205 |
| GO:0004970 | ionotropic glutamate receptor activity | MF | 1.54E-15 | 6.03E-14 | 38 |
| GO:0015276 | ligand-gated ion channel activity | MF | 1.53E-14 | 5.59E-13 | 44 |
| GO:0005515 | protein binding | MF | 1.3E-13 | 4.53E-12 | 1518 |
| GO:0008234 | cysteine-type peptidase activity | MF | 1.6E-12 | 4.88E-11 | 97 |
| GO:0043167 | ion binding | MF | 2.12E-11 | 5.45E-10 | 1663 |

| | | | | | |
|---|---|---|---|---|---|
| GO:0009733 | response to auxin | BP | 4.31E-11 | 1.08E-09 | 53 |
| GO:0010309 | acireductone dioxygenase [iron(II)-requiring] activity | MF | 5.75E-09 | 0.000000122 | 23 |
| GO:0022836 | gated channel activity | MF | 1.55E-08 | 0.000000307 | 53 |
| GO:0004523 | RNA-DNA hybrid ribonuclease activity | MF | 2.52E-08 | 0.000000483 | 21 |
| GO:0016757 | transferase activity, transferring glycosyl groups | MF | 2.95E-08 | 0.000000557 | 223 |
| GO:0000413 | protein peptidyl-prolyl isomerization | BP | 3.16E-08 | 0.000000581 | 56 |
| GO:0003755 | peptidyl-prolyl cis-trans isomerase activity | MF | 3.16E-08 | 0.000000581 | 56 |
| GO:0034062 | RNA polymerase activity | MF | 3.59E-08 | 0.000000656 | 115 |
| GO:0005216 | ion channel activity | MF | 0.000000343 | 0.00000561 | 76 |
| GO:0004888 | transmembrane signaling receptor activity | MF | 0.000000351 | 0.0000057 | 56 |
| GO:0051704 | multi-organism process | BP | 0.000000598 | 0.00000922 | 295 |
| GO:0003899 | DNA-directed RNA polymerase activity | MF | 0.0000015 | 0.0000217 | 87 |
| GO:0016702 | oxidoreductase activity | MF | 0.00000195 | 0.0000279 | 37 |
| GO:0008146 | sulfotransferase activity | MF | 0.00000217 | 0.0000308 | 15 |
| GO:0016725 | oxidoreductase activity, acting on CH or CH2 groups | MF | 0.00000217 | 0.0000308 | 15 |
| GO:0004650 | polygalacturonase activity | MF | 0.00000281 | 0.0000392 | 36 |
| GO:0007283 | spermatogenesis | BP | 0.00000298 | 0.0000408 | 15 |
| GO:0016717 | oxidoreductase activity | MF | 0.00000304 | 0.0000412 | 13 |
| GO:0009725 | response to hormone | BP | 0.00000544 | 0.0000712 | 57 |
| GO:0016168 | chlorophyll binding | MF | 0.00000681 | 0.000086 | 10 |
| GO:0018193 | peptidyl-amino acid modification | BP | 0.00000726 | 0.0000904 | 58 |
| GO:0016701 | oxidoreductase activity | MF | 0.00000742 | 0.0000915 | 45 |
| GO:0055114 | oxidation-reduction process | BP | 0.00000889 | 0.000108223 | 614 |
| GO:0004748 | ribonucleoside-diphosphate reductase activity | MF | 0.00000999 | 0.000119522 | 13 |
| GO:0015105 | arsenite transmembrane transporter activity | MF | 0.0000132 | 0.000155028 | 8 |
| GO:0015700 | arsenite transport | BP | 0.0000132 | 0.000155028 | 8 |
| GO:0000723 | telomere maintenance | BP | 0.0000232 | 0.000258538 | 23 |
| GO:0045300 | acyl-[acyl-carrier-protein] desaturase activity | MF | 0.0000253 | 0.000277704 | 9 |
| GO:0006784 | heme a biosynthetic process | BP | 0.0000332 | 0.000357347 | 11 |
| GO:0010033 | response to organic | BP | 0.0000634 | 0.000609258 | 59 |

| | | | | | |
|---|---|---|---|---|---|
| | substance | | | | |
| GO:0019013 | viral nucleocapsid | CC | 0.000075 | 0.000710877 | 27 |
| GO:0051213 | dioxygenase activity | MF | 0.000082 | 0.000771205 | 39 |
| GO:0016491 | oxidoreductase activity | MF | 0.0000934 | 0.000867116 | 619 |
| GO:0009289 | pilus | CC | 0.0000967 | 0.000888965 | 11 |
| GO:0003677 | DNA binding | MF | 0.000101952 | 0.000930985 | 668 |
| GO:0000981 | sequence-specific DNA binding RNA polymerase II transcription factor activity | MF | 0.000197423 | 0.001686293 | 13 |
| GO:0005173 | stem cell factor receptor binding | MF | 0.000338845 | 0.002766568 | 9 |
| GO:0030639 | polyketide biosynthetic process | BP | 0.000364424 | 0.002915395 | 8 |
| GO:0009318 | exodeoxyribonuclease VII complex | CC | 0.000364424 | 0.002915395 | 8 |
| GO:0038023 | signaling receptor activity | MF | 0.000425649 | 0.003361409 | 68 |
| GO:0047134 | protein-disulfide reductase activity | MF | 0.000427441 | 0.003361409 | 21 |
| GO:0009767 | photosynthetic electron transport chain | BP | 0.000529747 | 0.004073623 | 11 |
| GO:0004872 | receptor activity | MF | 0.000842189 | 0.006217866 | 74 |
| GO:0016984 | ribulose-bisphosphate carboxylase activity | MF | 0.000894494 | 0.00656909 | 5 |
| GO:0004867 | serine-type endopeptidase inhibitor activity | MF | 0.000989441 | 0.007190285 | 20 |
| GO:0007131 | reciprocal meiotic recombination | BP | 0.001046767 | 0.007528048 | 8 |
| GO:0006308 | DNA catabolic process | BP | 0.00188296 | 0.013067743 | 9 |
| GO:0010277 | chlorophyllide a oxygenase [overall] activity | MF | 0.002462613 | 0.016714457 | 8 |
| GO:0009611 | response to wounding | BP | 0.002931785 | 0.019527739 | 30 |
| GO:0009507 | chloroplast | CC | 0.00339389 | 0.022220376 | 11 |
| GO:0042627 | chylomicron | CC | 0.003642443 | 0.02308544 | 4 |
| GO:0015485 | cholesterol binding | MF | 0.003642443 | 0.02308544 | 4 |
| GO:0004420 | hydroxymethylglutaryl-CoA reductase (NADPH) activity | MF | 0.003642443 | 0.02308544 | 4 |
| GO:0015385 | sodium:proton antiporter activity | MF | 0.004268802 | 0.026451328 | 5 |
| GO:0010333 | terpene synthase activity | MF | 0.004479536 | 0.02739029 | 26 |
| GO:0007127 | meiosis I | BP | 0.006595219 | 0.03895389 | 9 |
| GO:0007276 | gamete generation | BP | 0.006701728 | 0.039323766 | 16 |
| GO:0005246 | calcium channel regulator | MF | 0.006813664 | 0.039323766 | 7 |

| | | | | | |
|---|---|---|---|---|---|
| | activity | | | | |
| GO:0004026 | alcohol O-acetyltransferase activity | MF | 0.006813664 | 0.039323766 | 7 |
| GO:0007016 | cytoskeletal anchoring at plasma membrane | BP | 0.006813664 | 0.039323766 | 7 |
| GO:0004656 | procollagen-proline 4-dioxygenase activity | MF | 0.006813664 | 0.039323766 | 7 |
| GO:0098519 | nucleotide phosphatase activity | MF | 0.008192751 | 0.046796452 | 34 |

**Table S20. Go enrichment results for contracted genes in *A. yangbiense*.**

| GO_ID | GO_Term | GO_Class | Pvalue | AdjustedPv | Gene_Number |
|---|---|---|---|---|---|
| GO:0043531 | ADP binding | MF | 8.09E-73 | 1.31E-69 | 48 |
| GO:0030246 | carbohydrate binding | MF | 2.16E-53 | 1.75E-50 | 44 |
| GO:0016747 | transferase activity | MF | 2.26E-27 | 1.23E-24 | 43 |
| GO:0004713 | protein tyrosine kinase activity | MF | 7.41E-19 | 3.01E-16 | 77 |
| GO:0016746 | transferase activity, transferring acyl groups | MF | 1.4E-16 | 4.55E-14 | 44 |
| GO:0006633 | fatty acid biosynthetic process | BP | 9.38E-15 | 2.31E-12 | 23 |
| GO:0004672 | protein kinase activity | MF | 9.93E-15 | 2.31E-12 | 92 |
| GO:0006631 | fatty acid metabolic process | BP | 3.97E-13 | 7.17E-11 | 24 |
| GO:0048544 | recognition of pollen | BP | 8.22E-12 | 1.03E-09 | 15 |
| GO:0006468 | protein phosphorylation | BP | 1.13E-11 | 1.31E-09 | 88 |
| GO:0016762 | xyloglucan:xyloglucosyl transferase activity | MF | 2.18E-10 | 2.08E-08 | 11 |
| GO:0048046 | apoplast | CC | 2.18E-10 | 2.08E-08 | 11 |
| GO:0016740 | transferase activity | MF | 2.27E-09 | 0.000000205 | 195 |
| GO:0030554 | adenyl nucleotide binding | MF | 2.45E-09 | 0.00000021 | 179 |
| GO:0016310 | phosphorylation | BP | 3.31E-09 | 0.000000269 | 92 |
| GO:0004523 | RNA-DNA hybrid ribonuclease activity | MF | 4.88E-09 | 0.000000378 | 8 |
| GO:0032559 | adenyl ribonucleotide binding | MF | 7.19E-09 | 0.000000532 | 175 |
| GO:0046394 | carboxylic acid biosynthetic process | BP | 1.22E-08 | 0.0000008 | 33 |
| GO:0017076 | purine nucleotide binding | MF | 1.23E-08 | 0.0000008 | 205 |
| GO:0032555 | purine ribonucleotide | MF | 1.64E-08 | 0.000000981 | 201 |

| | | | | | |
|---|---|---|---|---|---|
| | binding | | | | |
| GO:0032550 | purine ribonucleoside binding | MF | 1.69E-08 | 0.000000981 | 200 |
| GO:0016773 | phosphotransferase activity, alcohol group as acceptor | MF | 0.000000038 | 0.00000193 | 95 |
| GO:0016301 | kinase activity | MF | 6.35E-08 | 0.00000304 | 101 |
| GO:0043412 | macromolecule modification | BP | 7.38E-08 | 0.00000343 | 100 |
| GO:0006464 | cellular protein modification process | BP | 9.88E-08 | 0.00000423 | 95 |
| GO:0030042 | actin filament depolymerization | BP | 0.00000014 | 0.00000568 | 8 |
| GO:0009678 | hydrogen-translocating pyrophosphatase activity | MF | 0.000000166 | 0.00000638 | 7 |
| GO:0000166 | nucleotide binding | MF | 0.000000169 | 0.00000638 | 213 |
| GO:0005488 | binding | MF | 0.000000636 | 0.000023 | 577 |
| GO:0030036 | actin cytoskeleton organization | BP | 0.00000242 | 0.0000839 | 16 |
| GO:0004427 | inorganic diphosphatase activity | MF | 0.00000398 | 0.000124336 | 7 |
| GO:0003871 | 5-methyltetrahydropteroyltriglutamate-homocysteine S-methyltransferase activity | MF | 0.00000398 | 0.000124336 | 7 |
| GO:0006073 | cellular glucan metabolic process | BP | 0.00000553 | 0.000166566 | 16 |
| GO:0043168 | anion binding | MF | 0.00000564 | 0.000166628 | 208 |
| GO:0009086 | methionine biosynthetic process | BP | 0.00000592 | 0.000171751 | 7 |
| GO:0005618 | cell wall | CC | 0.00000647 | 0.00018468 | 16 |
| GO:0031429 | box H/ACA snoRNP complex | CC | 0.0000104 | 0.000271884 | 4 |
| GO:0005856 | cytoskeleton | CC | 0.0000118 | 0.000304912 | 53 |
| GO:0005515 | protein binding | MF | 0.0000137 | 0.000347269 | 229 |
| GO:0044264 | cellular polysaccharide metabolic process | BP | 0.0000181 | 0.000446959 | 17 |
| GO:0000160 | phosphorelay signal transduction system | BP | 0.0000284 | 0.00067969 | 11 |
| GO:0006796 | phosphate-containing compound metabolic process | BP | 0.0000776 | 0.001752006 | 115 |
| GO:0009067 | aspartate family amino | BP | 0.000079 | 0.001759818 | 8 |

| | | | | | |
|---|---|---|---|---|---|
| | acid biosynthetic process | | | | |
| GO:0016772 | Ttransferring phosphorus-containing groups | MF | 0.0000973 | 0.002080798 | 112 |
| GO:0005452 | inorganic anion exchanger activity | MF | 0.000134525 | 0.002804331 | 16 |
| GO:0043624 | cellular protein complex disassembly | BP | 0.000142659 | 0.002828816 | 9 |
| GO:0004602 | glutathione peroxidase activity | MF | 0.000230886 | 0.004416721 | 4 |
| GO:0015405 | P-P-bond-hydrolysis-driven transmembrane transporter activity | MF | 0.000242776 | 0.004537392 | 23 |
| GO:0005730 | nucleolus | CC | 0.000343401 | 0.006345118 | 5 |
| GO:0000148 | 1,3-beta-D-glucan synthase complex | CC | 0.000568618 | 0.010049703 | 4 |
| GO:0006075 | (1->3)-beta-D-glucan biosynthetic process | BP | 0.000568618 | 0.010049703 | 4 |
| GO:0043167 | ion binding | MF | 0.000834611 | 0.013939755 | 310 |
| GO:0044255 | cellular lipid metabolic process | BP | 0.001073147 | 0.017625625 | 26 |
| GO:0032787 | monocarboxylic acid metabolic process | BP | 0.001247597 | 0.020085075 | 28 |
| GO:0006026 | aminoglycan catabolic process | BP | 0.001417589 | 0.022598034 | 6 |
| GO:0007010 | cytoskeleton organization | BP | 0.001505952 | 0.023414636 | 25 |
| GO:0008154 | actin polymerization or depolymerization | BP | 0.001512015 | 0.023414636 | 9 |
| GO:1901363 | heterocyclic compound binding | MF | 0.001590837 | 0.024402834 | 350 |
| GO:0008171 | O-methyltransferase activity | MF | 0.001946788 | 0.028920648 | 6 |
| GO:0004553 | hydrolase activity, hydrolyzing O-glycosyl compounds | MF | 0.001956501 | 0.028920648 | 36 |
| GO:0035556 | intracellular signal transduction | BP | 0.002306731 | 0.033790497 | 27 |
| GO:0097159 | organic cyclic compound binding | MF | 0.002375685 | 0.03418463 | 350 |
| GO:0003682 | chromatin binding | MF | 0.002764538 | 0.039431048 | 21 |
| GO:0016712 | oxidoreductase activity, | MF | 0.003091261 | 0.043113172 | 11 |
| GO:0016798 | hydrolase activity, acting | MF | 0.003127957 | 0.043113172 | 37 |

| | | | | | |
|---|---|---|---|---|---|
| | on glycosyl bonds | | | | |
| GO:0004568 | chitinase activity | MF | 0.003261328 | 0.043113172 | 5 |
| GO:0006032 | chitin catabolic process | BP | 0.003261328 | 0.043113172 | 5 |
| GO:0003843 | 1,3-beta-D-glucan synthase activity | MF | 0.003413191 | 0.044756844 | 4 |

**Table S21. KEGG enrichment results for expanded genes in *A. yangbiense*.**

| MapID | MapTitle | Pvalue | AdjustedPv | Gene_Number |
|---|---|---|---|---|
| map04110 | Cell cycle | 1.25E-75 | 4.54E-74 | 344 |
| map04626 | Plant-pathogen interaction | 9.59E-58 | 2.79E-56 | 449 |
| map00903 | Limonene and pinene degradation | 5.44E-35 | 1.32E-33 | 103 |
| map00460 | Cyanoamino acid metabolism | 1.16E-28 | 2.6E-27 | 146 |
| map00624 | Polycyclic aromatic hydrocarbon degradation | 3.69E-24 | 7.66E-23 | 72 |
| map00363 | Bisphenol degradation | 1.23E-23 | 2.38E-22 | 67 |
| map00627 | Aminobenzoate degradation | 1.68E-20 | 2.88E-19 | 78 |
| map03020 | RNA polymerase | 2.75E-19 | 4.45E-18 | 97 |
| map00943 | Isoflavonoid biosynthesis | 2.32E-14 | 3.22E-13 | 39 |
| map00945 | Stilbenoid, diarylheptanoid and gingerol biosynthesis | 2.1E-12 | 2.28E-11 | 76 |
| map00950 | Isoquinoline alkaloid biosynthesis | 7.04E-11 | 6.4E-10 | 69 |
| map00942 | Anthocyanin biosynthesis | 9.15E-11 | 8.06E-10 | 27 |
| map03018 | RNA degradation | 1.23E-10 | 1.05E-09 | 154 |
| map00140 | Steroid hormone biosynthesis | 1.91E-10 | 1.59E-09 | 76 |
| map00940 | Phenylpropanoid biosynthesis | 5.3E-10 | 4.28E-09 | 172 |
| map00966 | Glucosinolate biosynthesis | 2.3E-09 | 1.72E-08 | 20 |
| map02010 | ABC transporters | 2.59E-08 | 0.000000171 | 107 |
| map00380 | Tryptophan metabolism | 0.000000104 | 0.000000605 | 55 |
| map00944 | Flavone and flavonol biosynthesis | 0.000000979 | 0.00000459 | 21 |
| map00350 | Tyrosine metabolism | 0.000002 | 0.00000925 | 69 |
| map00625 | Chloroalkane and chloroalkene degradation | 0.00000248 | 0.0000113 | 42 |
| map00340 | Histidine metabolism | 0.000003 | 0.0000132 | 36 |
| map00410 | beta-Alanine metabolism | 0.00000908 | 0.0000367 | 69 |
| map02020 | Two-component system | 0.0000119 | 0.0000469 | 29 |
| map00190 | Oxidative phosphorylation | 0.0000246 | 0.0000944 | 92 |
| map00071 | Fatty acid degradation | 0.0000464 | 0.000160842 | 42 |
| map00040 | Pentose and glucuronate interconversions | 0.0000534 | 0.000180588 | 125 |
| map03320 | PPAR signaling pathway | 0.000234972 | 0.000697724 | 32 |

| | | | | |
|---|---|---|---|---|
| map03008 | Ribosome biogenesis in eukaryotes | 0.000333736 | 0.000957837 | 80 |
| map00908 | Zeatin biosynthesis | 0.000711345 | 0.001916678 | 22 |
| map00053 | Ascorbate and aldarate metabolism | 0.000979026 | 0.002543719 | 50 |
| map00960 | Tropane, piperidine and pyridine alkaloid biosynthesis | 0.001214326 | 0.003020246 | 46 |
| map00901 | Indole alkaloid biosynthesis | 0.001561423 | 0.003786451 | 10 |
| map00480 | Glutathione metabolism | 0.001683074 | 0.004047722 | 64 |
| map00330 | Arginine and proline metabolism | 0.002505086 | 0.005564732 | 47 |
| map00240 | Pyrimidine metabolism | 0.003086864 | 0.006805132 | 104 |
| map00310 | Lysine degradation | 0.005026666 | 0.010374183 | 36 |
| map00591 | Linoleic acid metabolism | 0.010101875 | 0.019597637 | 16 |
| map00643 | Styrene degradation | 0.010512425 | 0.020125761 | 11 |
| map00730 | Thiamine metabolism | 0.01658878 | 0.029798363 | 26 |
| map00280 | Valine, leucine and isoleucine degradation | 0.024037992 | 0.040906758 | 43 |
| map00980 | Metabolism of xenobiotics by cytochrome P450 | 0.028726579 | 0.047228444 | 36 |

**Table S22. A summary of Drought-tolerant genes identified in *A. truncatum* and other published genomes.**

| Genes | *A.truncatum* | *A.yangbiense* | *A. thaliana* | *C. sinensis* | *M. oleifera* | *S. indicum* |
|---|---|---|---|---|---|---|
| APX | 16 | 16 | 20 | 18 | 13 | 22 |
| DHA | 4 | 1 | 6 | 4 | 3 | 5 |
| GCL | 4 | 8 | 3 | 2 | 2 | 4 |
| GLO | 24 | 23 | 18 | 16 | 11 | 11 |
| GR | 12 | 12 | 16 | 16 | 12 | 28 |
| GSH-Px | 5 | 4 | 8 | 5 | 5 | 6 |
| MDHAR | 3 | 3 | 5 | 3 | 2 | 3 |
| MSR | 8 | 3 | 4 | 2 | 1 | 3 |
| POD | 57 | 78 | 73 | 72 | 54 | 84 |
| AQP1 | 13 | 9 | 9 | 8 | 8 | 11 |
| LEA | 82 | 68 | 50 | 72 | 55 | 74 |
| LEW | 12 | 9 | 10 | 9 | 10 | 9 |
| NLM | 7 | 10 | 9 | 8 | 6 | 8 |
| OSM | 6 | 10 | 2 | 3 | 4 | 12 |
| PIP | 7 | 10 | 19 | 11 | 8 | 14 |
| SIP | 3 | 2 | 3 | 2 | 2 | 3 |

| | | | | | | |
|---|---|---|---|---|---|---|
| SOS1 | 1 | 1 | 2 | 1 | 0 | 1 |
| TIP | 14 | 9 | 11 | 9 | 9 | 12 |
| ACP | 3 | 4 | 4 | 3 | 1 | 4 |
| ALAT | 2 | 2 | 4 | 2 | 2 | 5 |
| AST | 7 | 7 | 5 | 6 | 5 | 4 |
| CHI | 2 | 2 | 3 | 1 | 2 | 2 |
| GOGAT | 1 | 1 | 2 | 1 | 0 | 1 |
| MDH | 6 | 5 | 5 | 5 | 3 | 5 |
| MS | 5 | 2 | 3 | 3 | 2 | 2 |
| NAD-ME | 2 | 2 | 2 | 2 | 1 | 2 |
| NIR | 1 | 1 | 1 | 1 | 1 | 2 |
| NR | 1 | 0 | 2 | 1 | 0 | 1 |
| PCK | 2 | 2 | 2 | 2 | 2 | 2 |
| PEPC1 | 6 | 4 | 3 | 3 | 4 | 4 |
| PPDK | 1 | 1 | 1 | 1 | 1 | 1 |
| SAH | 1 | 1 | 2 | 1 | 1 | 3 |
| SAMS | 1 | 5 | 4 | 4 | 4 | 4 |
| AFL1 | 0 | 0 | 1 | 0 | 0 | 0 |
| COMT | 9 | 14 | 2 | 28 | 2 | 3 |
| GRP | 14 | 9 | 31 | 11 | 12 | 17 |
| LOX | 5 | 12 | 6 | 10 | 8 | 15 |
| MDA1 | 1 | 1 | 1 | 1 | 1 | 1 |
| PAE | 10 | 12 | 14 | 9 | 7 | 11 |
| PAL | 2 | 8 | 4 | 4 | 5 | 8 |
| PGIP | 0 | 0 | 3 | 1 | 0 | 0 |
| PLD | 3 | 3 | 3 | 2 | 3 | 5 |
| RGP | 3 | 3 | 5 | 5 | 7 | 8 |
| XI | 4 | 5 | 10 | 5 | 2 | 8 |
| XTH | 25 | 10 | 33 | 31 | 30 | 36 |
| BADH | 1 | 1 | 2 | 1 | 0 | 2 |
| COD1 | 0 | 0 | 1 | 0 | 0 | 0 |
| DMT | 4 | 2 | 5 | 2 | 2 | 2 |
| P5CDH | 1 | 1 | 1 | 1 | 0 | 1 |
| P5CR | 2 | 2 | 1 | 1 | 1 | 1 |
| P5CS | 1 | 1 | 2 | 2 | 2 | 2 |
| PEAMT | 1 | 1 | 1 | 1 | 0 | 2 |
| ProDH | 1 | 1 | 2 | 1 | 1 | 2 |
| SPS | 5 | 4 | 6 | 5 | 2 | 5 |
| SUS | 12 | 6 | 9 | 7 | 7 | 7 |
| TPS | 10 | 8 | 10 | 8 | 11 | 10 |

**Table S23. A summary of drought-tolerant transcription fators identified in *A. truncatum* and other published genomes.**

| Genes | *A. truncatum* | *A.yangbiense* | *A. thaliana* | *C. sinensis* | *M. oleifera* | *S. indicum* |
|---|---|---|---|---|---|---|
| ADH | 6 | 5 | 7 | 9 | 10 | 8 |
| AREB | 0 | 1 | 5 | 2 | 4 | 1 |
| COR | 0 | 0 | 2 | 0 | 0 | 0 |
| DREB | 6 | 9 | 9 | 6 | 3 | 7 |
| EIL1 | 4 | 3 | 3 | 4 | 2 | 5 |
| HARDY | 5 | 5 | 5 | 9 | 1 | 4 |
| KIN | 43 | 33 | 47 | 37 | 31 | 38 |
| MYB | 19 | 16 | 28 | 19 | 15 | 19 |
| NAC3 | 15 | 3 | 27 | 24 | 3 | 18 |
| NAC6 | 30 | 7 | 50 | 34 | 7 | 42 |
| RD22 | 1 | 1 | 1 | 1 | 1 | 1 |
| RD29B | 0 | 0 | 1 | 0 | 0 | 0 |
| WRKY | 2 | 2 | 11 | 5 | 7 | 2 |
| bZIP23 | 1 | 1 | 3 | 2 | 2 | 2 |

**Table S24. The gene copy number of VLCFA biosynthesis in 12 plants.**

| GeneID | *A.truncatum* | *A.yangbiense* | *A.thaliana* | *C. sinensis* | *G. max* | *G. raimondii* | *H. annuus* | *M. oleifera* | *O. europae* | *S. indicum* | *S. lycopersicum* | *X. sorbifolium* |
|---|---|---|---|---|---|---|---|---|---|---|---|---|
| ACC | 1 | 1 | 2 | 0 | 2 | 2 | 2 | 0 | 1 | 1 | 1 | 1 |
| a-CT | 1 | 2 | 1 | 1 | 3 | 2 | 3 | 0 | 3 | 3 | 1 | 0 |
| BCCP | 2 | 2 | 2 | 2 | 5 | 4 | 2 | 2 | 4 | 1 | 2 | 0 |
| BC | 1 | 1 | 1 | 1 | 2 | 2 | 2 | 0 | 1 | 1 | 1 | 1 |
| b-CT | 0 | 1 | 1 | 0 | 0 | 0 | 0 | 0 | 0 | 0 | 1 | 0 |
| ECR | 3 | 2 | 1 | 1 | 2 | 2 | 3 | 1 | 2 | 1 | 1 | 1 |
| ER | 3 | 3 | 2 | 2 | 5 | 4 | 5 | 4 | 4 | 4 | 5 | 2 |
| FATA | 2 | 1 | 2 | 1 | 1 | 3 | 1 | 0 | 2 | 1 | 1 | 1 |
| FATB | 1 | 1 | 1 | 1 | 4 | 4 | 3 | 1 | 1 | 1 | 1 | 2 |
| HAD | 3 | 3 | 3 | 1 | 7 | 3 | 3 | 2 | 2 | 2 | 3 | 2 |
| HCD | 2 | 2 | 2 | 2 | 3 | 2 | 3 | 1 | 3 | 3 | 2 | 2 |
| KAR | 3 | 3 | 7 | 4 | 6 | 4 | 9 | 2 | 4 | 8 | 7 | 2 |
| KASIII | 1 | 1 | 1 | 0 | 3 | 2 | 3 | 0 | 2 | 2 | 2 | 0 |
| KASII | 1 | 1 | 2 | 5 | 7 | 8 | 9 | 3 | 8 | 6 | 5 | 0 |
| KASI | 2 | 2 | 2 | 3 | 8 | 7 | 9 | 2 | 8 | 6 | 5 | 1 |
| KCR | 1 | 4 | 2 | 2 | 3 | 3 | 4 | 2 | 4 | 3 | 2 | 2 |
| KCS | 28 | 22 | 21 | 15 | 31 | 26 | 33 | 19 | 28 | 21 | 17 | 13 |
| LACS | 9 | 7 | 9 | 7 | 20 | 12 | 13 | 5 | 11 | 9 | 9 | 2 |
| MCMT | 1 | 1 | 1 | 1 | 2 | 1 | 2 | 1 | 0 | 1 | 1 | 1 |
| SAD | 3 | 10 | 7 | 6 | 5 | 8 | 2 | 6 | 5 | 7 | 7 | 4 |

**Table S25. Expression levels significantly up-regulated between 85 DAF and 100DAF in the fatty acid elongation stage of the VLCFA biosynthesis pathway.**

| GENE_ID | readcount_100DAF | readcount_85DAF | log2FoldChange | p-value |
|---|---|---|---|---|
| Atru.chr4.2307 | 117.7472816 | 4.410290476 | 4.7387 | 3.15E-16 |
| Atru.chr4.2308 | 8356.115812 | 439.3283629 | 4.2495 | 1.12E-87 |
| Atru.chr4.2311 | 4979.706479 | 512.3433797 | 3.2809 | 3.32E-10 |

**Table S26. The transcription factors (TF)/transcription regulatory factors (TR) in black module of WGCNA analysis.**

| Module | TF/TR | Family | Gene ID |
|---|---|---|---|
| black | TF | MYB-related | Atru.chr1.3799, Atru.chr8.1841, Atru.chr3.3572 |
| | TF | Alfin-like | Atru.chr1.3876 |
| | TF | Trihelix | Atru.chr11.2229.1 |
| | TF | WRKY | Atru.chr12.201, Atru.chr12.202, Atru.chr9.2017, Atru.chr4.3047 |
| | TF | C3H | Atru.chr13.328 |
| | TF | LOB | Atru.chr3.3711 |
| | TF | B3 | Atru.chr5.2849.2 |
| | TF | NAC | Atru.chr5.3491 |
| | TF | bZIP | Atru.chr5.613 |
| | TF | ERF | Atru.chr5.683 |
| | TR | MBF1 | Atru.chr7.1330 |
| | TF | ERF | Atru.chr7.2513 |

**Table S27. List of *KCS* gene-specific primer (5' - 3') used for real-time RT-PCR.**

| gene name | Forward primer | Reverse primer |
|---|---|---|
| Atru.chr4.2308 | TTGGGCAAATGACTTACGGG | AATCATTGCCGATAAAGACG |
| Atru.chr4.2307 | TCCAAACTTCTTCCTCTCTG | AGACAAAGGCAACAGAGCAT |
| Atru.chr4.2311 | TTCATCCCAACTACTATGCG | TCGGTTGGAGAGGAGGATGG |
| Atru.chr4.2309 | GTTGATGCAGGTTCCTCATA | CTAGGCTTCACTCCAGTTTT |
| Atru.chr4.2822 | TGGATGCGAGAAAGGAGACA | CTGATAAAGACGGCGTTGGA |
| Actin | ATCATGTTTGAGACCTTCAACAC | GATCTTCATGAGGTAGTCAGTCAGGT |

**Table S28. List of *LEA* gene-specific primer (5' - 3') used for real-time RT-PCR.**

| Gene name | Forward primer | Reverse primer |
|---|---|---|
| LEA1-Atru.chr6.1738 | AAGCAGCAGCGAGGACAGAG | GGTCACCGCCAGATGGGATT |
| LEA2-Atru.chr3.1961 | GATTACGAGGTTTGGCTGTC | TTGTTCTTCTTGAGGCGAGT |
| LEA2-Atru.chr10.2344 | CATGCCCTTTCCTTCCCTAA | TCATCGTGCTTGACTTCCTG |
| LEA2-Atru.chr1.3277 | ATCATCACAGTCATCATCCTCCTC | CGTGTCCTAGCTTCCTCCCT |
| LEA3-Atru.chr12.2033 | CTCCTCCTACGGCTCAACTC | CAGGCTTTCTCCTCCAACAC |
| LEA4-Atru.chr6.2817 | AACAGCAAGGTCGTCCACAG | CAGTTCCAGCACCAAGTTTA |
| LEA5-Atru.chr12.68 | ATGTTAGTGTTACGGAGGTTTGA | CCTAGCCTTGGTGTCGAGTT |
| SMP-Atru.chr5.1467 | TCTCACCGACATCACCAGAG | AGTTGCCACATTAGGCTTTG |
| Dehydrin-Atru.chr5.3081 | GGATGATGGGCAAGGTGGAA | AGGCAACGGTTGTGGTCTGT |
| Dehydrin-Atru.chr3.677 | TGGGATACGGAAGAAGAAGG | GACAGGAGTAGTGGTGGTTGC |
